# Design, Synthesis, Spectroscopic Inspection, DFT and Molecular Docking Study of Metal Chelates Incorporating Azo Dye Ligand for Biological Evaluation

**DOI:** 10.3390/ma16030897

**Published:** 2023-01-17

**Authors:** Mohamed Ali Ibrahim Al-Gaber, Hany M. Abd El-Lateef, Mai M. Khalaf, Saad Shaaban, Mohamed Shawky, Gehad G. Mohamed, Aly Abdou, Mohamed Gouda, Ahmed M. Abu-Dief

**Affiliations:** 1 Department of Chemistry, College of Science, King Faisal University, Al-Ahsa 31982, Saudi Arabia; 2 Department of Chemistry, Faculty of Science, Sohag University, Sohag 82534, Egypt; 3 Chemistry Department, Faculty of Science, Mansoura University, Mansoura 35516, Egypt; 4 Chemistry Department, Faculty of Science, Cairo University, Giza 12613, Egypt; 5 Nanoscience Department, Basic and Applied Sciences Institute, Egypt-Japan University of Science and Technology, New Borg El Arab, Alexandria 21934, Egypt; 6 Chemistry Department, College of Science, Taibah University, Medinah 42344, Saudi Arabia

**Keywords:** azo dye, complexes, antimicrobial activities, docking, molecular geometries

## Abstract

A new heterocyclic azo dye ligand (L) was synthesized by the combination of 4-amino antipyrine with 4-aminophenol. The new Cr(III), Mn(II), Fe(III), Co(II), Ni(II), Cu(II), Zn(II), and Cd(II) complexes were synthesized in excellent yields. The metal chelate structures were elucidated using elemental analyses, FT-IR, ^1^H-NMR, mass, magnetic moment, diffused reflectance spectral and thermal analysis (TG-DTG), and molar conductivity measurement. According to the FT-IR study, the azo dye ligand exhibited neutral tri-dentate behavior, binding to the metal ions with the azo N, carbonyl O, and protonated phenolic OH. The ^1^H-NMR spectral study of the Zn(II) complex supported the coordination of the zo dye ligand without proton displacement of the phenolic OH. Diffused reflectance and magnetic moment studies revealed the octahedral geometry of the complexes, as well as their good electrolytic nature, excepting the Zn(II) and Cd(II) complexes, which were nonelectrolytes, as deduced from the molar conductivity study. The theoretical calculations of optimized HOMO–LUMO energies, geometrical parameters, electronic spectra, natural atomic charges, 3D-plots of MEP, and vibrational wavenumbers were computed and elucidated using LANL2DZ and 6-311G (d, p) basis sets of density functional theory (DFT) with the approach of B3LYP DFT and TD-DFT methods. The ligand and complexes have been assayed for their antimicrobial activity and compared with the standard drugs. Most of the complexes have manifested excellent antimicrobial activity against various microbial strains. A molecular docking investigation was also performed, to acquire more information about the binding mode and energy of the ligand and its metal complexes to the *Escherichia coli* receptor using molecular docking. Altogether, the newly created ligand and complexes showed positive antibacterial effects and are worth future study.

## 1. Introduction

The most popular synthetic dyes used in industry are azo dyes. The latter are aromatic substances that include the diazinyl (N=N) groups [1]. Azo dyes are frequently involved in many industries, including food and textile, paper printing, and cosmetics [2,3]. On the other hand, coordination chemistry plays an important role in enhancing the biomedical application of ligands [4,5,6,7,8,9,10]. The azo group possesses excellent donor properties and is important in coordination chemistry. Therefore, chemists have recently become interested in developing and physicochemical characterization of first-row transition metal chelates with a wide range of azo dye ligands [11,12]. Furthermore, azo dye-based metal chelates have contributed significantly to the modern coordination chemistry revolution, due to their interesting properties and diverse applications [13].

Furthermore, their facile synthetic protocol and structural diversity allows access to a wide range of metal chelates [14,15]. Moreover, simple structural modifications (e.g., incorporation of bioactive heterocycles) and chelation with metal ions are usually associated with characteristic alterations and enhancement of their biological properties, including the antioxidant and antiproliferative activities [16], as well as their DNA binding affinities [17,18]. For instance, coordination is usually associated with bathochromic shift, making the color duller. Besides this, oxidation resistance and light fastness properties are increased, whereas aqueous solubility is lowered.

The current study describes the synthesis of new azo dye ligands derived from 4-amino-antipyrine with 4-aminophenol. The resulting novel azo dye ligand coordination was investigated with metal ions such as CrIII, MnII, FeIII, CoII, NiII, CuII, ZnII, and CdII ions. Their corresponding composition was elucidated using various spectral methods, such as IR and ^1^H NMR. The latter aided the verification of the coordination manner. Furthermore, DFT calculations supported the structural elucidation. Likewise, the antimicrobial properties of the azo dye ligand and its metal chelates were also assessed. Moreover, the antimicrobial activity was confirmed using molecular docking.

## 2. Materials and Methods

### 2.1. Starting Materials, Reagents, and Methods

All chemicals and methods employed in the preparation and characterization of the compounds under inspection are supplied in the supporting information.

### 2.2. Azo Dye Ligand (L) Preparation

4-Amino-antipyrine (3 g, 14.85 mmol) was dissolved in EtOH (40 mL) and put in a 100 mL flask. HCl solution (4 mL) was added at 0–5 °C while stirring. Sodium nitrite solution (5 g, 72.4 mmol, 10 mL H_2_O) was added drop-by-drop over 10 min, and the resulting solution was then agitated for further 20 min at 0–5 °C. Stirring was continued for an additional hour, and the mixture was poured into the 2,4-diaminophenol coupling component (1.61 g, 14.85 mmol) in EtOH (25 mL) and an aqueous catalyst solution of 4 g CH_3_COONa. The produced compound was sterilized through filtration, washed with H_2_O, and vacuum-dried at 298 K.

Yield 84%; m.p. = 124 °C; brownish-red solid. Analysis Calcd for C_17_H_17_N_5_O_2_(%):C, 63.16; H, 5.26; N, 21.67. Found (%): C, 63.10; H, 5.02; N, 21.48. IR (cm^−1^):ν(OH) 3433; carbonyl group ν(C=O) 1639; azo ν(N=N) 1620.^1^H/ NMR (400 MHz/d_6_ -DMSO/δ, ppm): 7.37–7.72 (m, 8H, Ar H), 3.43 (s, 3H, CH_3_-N), 2.54 (s, 3H, CH_3_), 6.90 (m, 2H, NH_2_), 10.00(s, 1H, OH). 

### 2.3. Metal Chelates Preparation

In EtOH, azo dye ligand (L) was dissolved (0.4 g, 1.2383 mmol). The identical molar ratio (1:1) of the following ethanolic metal salt solutions was added to the solution of azo ligand: 0.3300 g Cr(III), 0.2004 g Mn(II), 0.3347 g Fe(III), 0.293 g Co(II), 0.294 g Ni(II), 0.211 g Cu(II), 0.169 g Zn(II), and 0.227 g Cd(II). For 2–3 hours, the mixture was reflux heated while stirring. The precipitates were removed by filtering and then dried over dehydrated CaCl_2_ in a vacuum desiccator after being washed with EtOH and diethyl ether.

[Cr(L)Cl_2_(H_2_O)]Cl Yield = 73%; m.p. = 235 °C; solid brown. Analysis Calcd for Cr(C_17_H_19_Cl_3_N_5_O_3_) (%): C, 40.84; H, 3.80; N, 14.01; Cr, 10.41; Found (%): C, 40.74; H, 3.70; N, 13.81; Cr, 10.23. μ_eff_ /BM 3.81; Λ_m_ /Ω^−1^ mol^−1^ cm^2^ 56.4. IR /cm^−1^: carbonyl group ν(C=O) 1628; ν(OH) 3402; azo ν(N=N) 1610; ν(M-O) 551; ν(M-N) 435. λ_max_ (nm): 241 (π–π*), 349 (n–π*). Ref. S.: 17,361, 19,436 and 22,624 cm^−1^ assignable to ^4^A_2_g(F) to ^4^T_1_g(F), ^4^A_2_g(F) to ^4^T_2_g(F) and ^4^A_2_g(F) to ^4^T_1_g(P) transitions, respectively.

[Mn(L)Cl_2_(H_2_O)]2H_2_O. Yield = 85%; solid yellowish brown; m.p. = 205 °C. Analysis Calcd for Mn (C_17_H_23_Cl_2_N_5_O_5_) (%): C, 40.56; H, 4.57; N, 13.92; Mn, 10.93; Found (%): C, 40.13; H, 4.50; N, 13.72; Mn, 10.68. μ_eff_ /BM 5.51; Λ_m_/Ω^−1^ cm^2^ mol^−1^ 46.4. IR: carbonyl group ν(C=O) 1623; ν(OH) 3420; azo ν(N=N) 1615, ν(M-O) 597; ν(M-N) 436. λ_max_ (nm): 219 (π–π*), 368 (n–π*). Ref. S.: 19,950, 21,260 and 25,478 cm^−1^ assignable to ^6^A_1_g → T_2_g(F), ^6^A_2_g(G) → ^5^T_2_g(F) and charge transfer transitions, respectively.

[Fe(L)Cl_2_(H_2_O)]Cl.H_2_O Yield = 88%; solid brown; m.p. = 215 °C. Analysis calculated for Fe(C_17_H_21_Cl_3_N_5_O_4_) (%): C, 39.12; H, 4.03; N, 13.42; Fe, 10.74; Found (%): C, 39.02; H, 4.01; N, 13.38; Fe, 11.05. μ_eff_ /BM 5.05; Λ_m_/Ω^−1^ cm^2^ mol^−1^ 93.6. IR: carbonyl group ν(C=O) 1627; ν(OH) 3407; azo ν(N=N) 1614; ν(M-O) 572; ν(M-N) 433. λ_max_ (nm): 219 (π–π*), 365 (n–π*). Ref. S.: 13,280, 18,580 and 27,625 cm^−1^ assignable to ^3^A_2_g(F) → ^3^T_2_g(F), ^3^A_2_g(F) → ^3^T_1_g(F) and ^3^A_2_g(F) → ^3^T_1_g(P) transitions, respectively.

[Co(L)Cl(H_2_O)]Cl.H_2_O. Yield = 83%; solid faint pink; m.p. = 250 °C. Analysis calculated for Co(C_17_H_21_Cl_2_N_5_O_4_) (%): C, 41.72; H, 4.29; N, 14.32; Co, 12.07; Found (%): C, 41.64; H, 4.20; N, 14.25; Co, 12.06. μ_eff_: 5.18; Λ_m_/Ω^−1^ cm^2^ mol^−1^ 36.4. IR: carbonyl group ν(C=O) 1634; ν(OH) 3421; azo ν(N=N) 1618; ν(M-O) 513; ν(M-N) 434. λ_max_ (nm): 221 (π–π*), 369 (n–π*). Ref. S.: 10,690, 15,187 and 25,025 cm^−1^ assignable to ^4^T_1_g to ^4^T_2_g(F), ^4^T_1_g to ^4^A_2_g(F) and ^4^T_1_g to ^4^T_1_g(p) transitions, respectively.

[Ni(L)Cl(H_2_O)_2_]Cl. Yield= 83%; solid faint green; m.p. 240 °C. Analysis calculated for Ni(C_17_H_21_Cl_2_N_5_O_4_) (%): C, 41.72; H, 4.29; N, 14.32; Ni, 12.07; Found (%): C, 41.66; H, 4.15; N, 14.08; Ni, 12.63. μ_eff_: 3.76; Λ_m_/Ω^−1^ cm^2^ mol^−1^ 83.3. IR: carbonyl group ν(C=O) 1626; ν(OH) 3416; azo ν(N=N) 1610; ν(M-O) 529; ν(M-N) 433. λ_max_ (nm): 261 π–π*, 369 n–π*. Ref. S.: 13,455, 16,253 and 22,056 cm^−1^ assignable to ^3^A_2_g to ^3^T_2_g,^3^A_2_g to ^3^T_1_g(F) and ^3^A_2_g to ^3^T_1_g(P) transitions, respectively.

[Cu(L)Cl(H_2_O)_2_]Cl. Yield= 89%; solid yellowish brown; m.p. 240 °C. Analysis calculated for Cu(C_17_H_21_Cl_2_N_5_O_4_) (%): C, 39.88; H, 4.50; N, 13.69; Cu, 12.41; Found (%): C, 39.66; H, 4.42; N, 13.30; Cu, 12.32. μ_eff_: 1.79; Λ_m_/Ω^−1^ cm^2^ mol^−1^ 88.3. IR: carbonyl group ν(C=O) 1640; ν(OH) 3434; azo ν(N=N) 1614; ν(M-O) 527; ν(M-N) 435. λ_max_ (nm): 269 (π–π*), 368 (n–π*). Ref. S.: 18,950, 21,260, and 24,478 cm^−1^ assignable to ^6^A_1_g→ T_2_g, ^6^A_1_g→ ^5^T_1_g(F), and charge transfer transitions, respectively.

[Zn(L)Cl_2_(H_2_O)]2H_2_O. Yield = 89%; solid brownish white; m.p. = 185 °C. Analysis calculated for Zn(C_17_H_23_Cl_2_N_5_O_5_) (%): C, 39.77; H, 4.48; N, 13.65; Zn, 12.67; Found (%): C, 39.35; H, 4.33; N, 13.58; Zn, 12.86. μ_eff._ diamagnetic; Λ_m_/Ω^−1^ cm^2^ mol^−1^ 39.4. IR: carbonyl group ν(C=O) 1645; ν(OH) 3431; azo ν(N=N) 1622; ν(M-O) 576; ν(M-N) 435 λ_max_ (nm): 221 (π–π*), 369 (n–π*). ^1^H NMR: 7.29–7.49 (m, 8H, Ar H), 3.41 (s, 3H, CH_3_-N), 2.52 (s, 3H, CH_3_), 6.91 (m, 2H, NH_2_):10.20(s, 1H, OH). λ_max_ (nm): 221 (π–π*), 368 n–π*.

[Cd(L)Cl_2_(H_2_O)]. Yield= 83%; solid brownish white; m.p. = 145 °C. Analysis calculated for Cd(C_17_H_19_Cl_2_N_5_O_3_) (%): C, 38.93; H, 3.63; N,13.36; Cd, 21.37; Found (%): C, 39.72; H, 3.60; N, 13.25; Cd, 21.43. μ_eff_. diamagnetic; Λ_m_ /Ω^−1^ cm^2^ mol^−1^ 31.2. IR: carbonyl group ν(C=O) 1629; ν(OH) 3439; azo ν(N=N) 1610; ν(M-O) 577; ν(M-N) 430 λ_max_ (nm): 241 (π–π*), 368 (n–π*). ^1^H NMR: 7.33–7.72 (m, 8H, Ar H), 3.40 (s, 3H, CH_3_-N), 2.50 (s, 3H, CH_3_), 6.91 (m, 2H, NH_2_), 9.40(s, 1H, OH).

### 2.4. Spectrophotometric Measurements

To study UV-Vis spectra in the region of 200:700 nm, stock solutions of metal chelates of 1 × 10^−4^ Mol/L were created by dissolving an exact quantity of the metal chelates in DMF.

### 2.5. DFT Calculations

The Gaussian 09 was used for all quantum chemistry computations and visualizations [19]. The DFT approach was used to perform geometry optimizations at the B3LYP level, utilizing the 6-311G(d, p) basis set for O, N, C, and H atoms [20]. LANL2DZ was used for the Co, Ni, Cu, Mn, Fe, Cr, Zn, and Cd atoms [19]. Using the optimized structures, frontier molecular orbitals (LUMOs and HOMOs) and MEP have been estimated [21]. Chemical hardness (η) and softness (σ), and other additional chemical descriptors that provide insight into the reactivity of molecules, were calculated [22].

### 2.6. Pharmacological Studies

#### 2.6.1. Anti-Pathogenic Activity

A filter paper disk (5 mm) was added to 100 mL flasks that comprised 15 mL of the test solution’s working concentration (100 mg/mL). All flasks were autoclaved at 121 °C for 20 min. Four bacterial strains were used, namely: *Bacillus subtilis* (*B. subtilis*) and *Staphylococcus aureus (S. aureus*) Gram(+ve), and *Salmonella sp.* and *Escherichia coli* (*E. coli*) Gram(-ve) bacteria. Furthermore, *Aspergillus* *fumigatus* (*A. fumigatus*) and *Candida albicans* (*C. albicans*) fungal strains were also used. All microbes were inoculated on the surfaces of LB agar media utilizing the diffusion agar technique (agar plates). All the chemicals were distributed among four evenly spaced locations in the inoculated Petri plates, each 2 cm from the center. A reference solvent for antipathogenic activity was DMSO. Clear or inhibitory zones were seen around every disk after these incubations at 25 °C for 48 h. The experiment’s control flask was created to run under the same conditions previously explained for each microbe, but only using a DMSO solution. The antibacterial activity was estimated by subtracting the diameter of the inhibition zone created by the DMSO treatment from the diameter obtained in each experiment [23,24,25].

Amikacin and ketoconazole are standard drugs used as references for their antifungal and antibacterial properties, respectively. The data shown are the mean values from all experiments carried out in triplicate.

#### 2.6.2. Docking for the Inspected Molecules

Molecular docking is frequently used to deduce structure-based activity relationships, as it enables the prediction of the confirmation of small-molecule ligands’ binding to the desired binding location. The binding nature of the metal chelates was predicted using MOE software. In addition, a file containing the crystal structure of the *E. coli* receptor’s active site (PDB ID: 3t88) was retrieved from the data bank of the protein.

## 3. Results & Discussion

### 3.1. Structural Inspection of the Azo Dye Ligand

**Figure 1:**
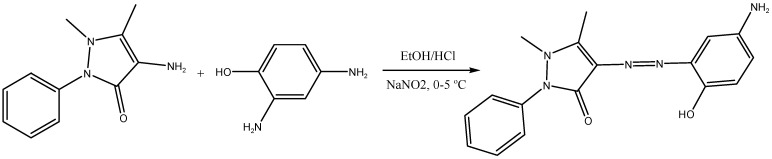
The proposed structure of azo dye ligand (L).

The coupling of 4-amino-antipyrine with 4-aminophenol led to the formation of the free ligand L, as shown in Figure 1. The free ligand L is soluble in ethanol, DMF, and DMSO solvents. The IR spectrum of the L ligand demonstrated in the experimental section (Figure S1) revealed three bands that were assigned to the stretching vibrations of OH, C=O, and N=N functions: a broad band at 3433 cm^−1^; a medium band at 1639 cm^−1^; and a faint band at 1620 cm^−1^. Unfortunately, the NH_2_ band is not very distinct, due to the overlap with the OH bands in the same area [26].

**Figure 2:**
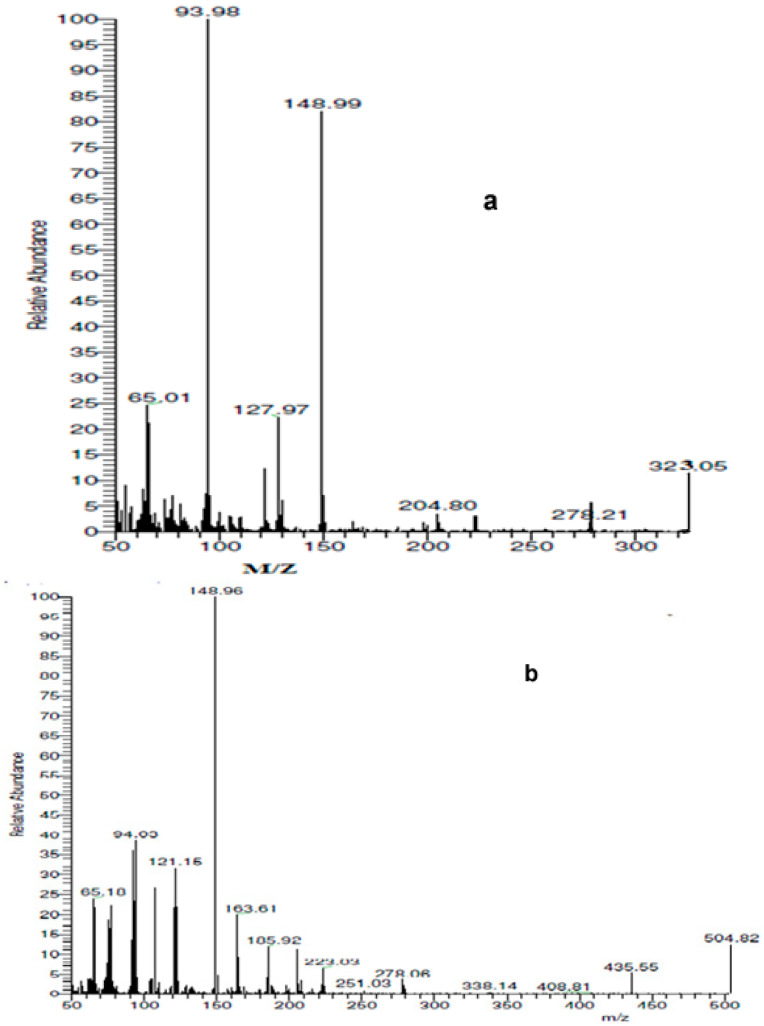
Mass spectra of (**a**) azo dye ligand (L), (**b**) Cd L complex.

The ligand’s ^1^H NMR spectra revealed a single signal at 10 ppm that is associated with the OH proton. The aromatic ring protons appeared multiple times between 7.37 and 7.72 ppm, followed by amino group signals at 6.9 ppm and the two methyl group signals at 3.43 ppm (Figure S2). When deuterated solvent was added, the signals from the OH and NH_2_ groups vanished from the ligand’s ^1^H NMR spectra, confirming their positions. The major molecular ion (M^+^) peak was found at *m*/*z* = 323 amu, which matches the elemental analysis results. The rest molecular ion peaks with their respective intensities are provided in Figure 2.

### 3.2. Structural Identification of Metal Chelates under Inspection

#### 3.2.1. C, H, and N percent in the compounds under investigation 

The experimental section and the complex’s molecular formulae show the elemental analyses (C, H, and N). The metal-ligand ratio in all the metal chelates examined was found to be 1:1. The metal chelates have high melting (decomposition) points and are stable in the presence of air. All the metal chelates are entirely insoluble in water but readily soluble in EtOH, DMF, and DMSO.

#### 3.2.2. Evaluation of Molar Conductance 

The molar conductivities were scanned at 27 °C using 10^−3^ mol/L of each metal chelate in DMF as solvent. The molar conductivity findings were outlined in the experimental section. The Mn^II^, Co^II^, Zn^II,^ and Cd^II^ complexes had molar conductivities of 36.4, 36, 39.4, and 11.2 Ω^−1^mol^−1^cm^2^, respectively, showing that they were non-electrolytes and non-ionic [27,28]. On the other hand, the molar conductivity values of the chelates of Cr(III), Ni(II), Cu(II), and Fe(III) were found to be 56.3, 83.3, 88.3, and 93.4 Ω^−1^mol^−1^cm^2^, respectively, revealing their ionic character and confirming that they were 1:1 electrolytes.

#### 3.2.3. IR Spectral Studies

The metal chelates’ infrared spectral bands are listed in the experimental section and Supplementary Figure S1. The coordination sites incorporated in chelation were deduced by comparing the IR spectra of the free ligand and its metal chelates. All produced metal complexes’ characteristic IR spectra assignments contrasted with the matching free ligand. Because the metal complexes do not have the same absorption bands as the free azo dye ligand, this suggests that the ligand and metal ion are coordinated. 

The band seen in the L spectrum at 3433 cm^−1^ strongly suggests that OH is present in L [29] while, in the spectra of the metal chelates, it was moved to 3402–3439 cm^−1^. The absorption band confirmed the presence of the carbonyl group in the free ligand at 1639 cm^−1^, which was shifted to 1623–1645 cm^−1^ in the metal chelates IR spectra as a result of the complex formation [30,31]. The band at 1620 cm^−1^ was attributed to the azo group’s stretching vibration in the free ligand and was shifted to 1610–1622 cm^−1^ in the metal chelates spectra [32] due to complexation of the ligand. The OH, C=O, and N=N groups’ shifts in band positions confirmed their participation in the bonding to the metal ions. 

A new band attributed to the symmetric and asymmetric vibration of coordinated water molecules occurred at 941–975 and 835–863 cm^−1^. It was determined that v(M-O) and (M-N) were responsible for the weak bands’ frequencies in the ranges of 551–577 cm^−1^ and 430–436 cm^−1^, respectively [33,34,35]. These bands did not arise in azo ligand; they were seen in metal complexes. The ligand demonstrates neutral tridentate behavior with NOO donor sites, according to the aforementioned information.

#### 3.2.4. Spectral Studies via ^1^H NMR

By considering the variations in the ^1^H NMR spectra of Cd^II^ and Zn^II^ chelates compared to the free ligand (Supplementary Figure S1), significant evidence for the structure of complexes was obtained. The phenyl group in the free ligand caused a cluster of multiple signals to appear between 7.37 and 7.72 ppm (m, 8H, Ar H). The phenolic-OH group in the ligand was responsible for the signal at 10 ppm (s, 1H, OH) [36], while the signals at 3.43 ppm (s, 6H, CH_3_) and 6.9 ppm (s, 2H, NH_2_) were attributed to the methyl and amino groups, respectively. The ^1^H NMR spectra of the Zn^II^ and Cd^II^ chelates revealed signals for the methyl, aromatic, and phenolic OH groups at 3.41 and 3.40 ppm (s, 6H, CH_3_), 7.29–7.49 and 7.29–7.72 ppm (m, 8H, Ar-H), and 10.20 and 9.40 ppm (s, 1H, OH), respectively. The Zn^II^ and Cd^II^ chelates signals at 6.90 and 6.91 ppm (s, 2H, NH_2_) belong to the amino group. The change in the location of the OH band demonstrated that phenolic groups’ oxygen atoms were coordinated with the metal ions. Additionally, it showed no proton displacement between the phenolic OH group and the metal ions.

#### 3.2.5. Mass Spectral Investigations

The hypothesized structures in Figure 2 are supported by the mass spectral data of the free ligand and its Cd^II^ azo dye complex, which are consistent with the molecular ion fragments. The molecular ion peak at *m*/*z* 504.82 amu in the Cd(II) chelate’s recorded mass spectrum was in good agreement with the expected values and corresponded to the molecular weight of the relevant compounds (M-H_2_O).

#### 3.2.6. UV–Vis Absorption Studies

Several factors affect the UV/Vis spectra of azo dye and its chelates. These include chemical composition, such as chromophores, substituted groups, azo groups number, cations, pH levels, solvents, and more, which frequently affect the UV/Vis spectra of these dyes [37]. The same solvent was used as a blank to measure the UV/Vis spectra of the free azo ligand (L) (1 × 10^−4^ M) and its chelates (1 × 10^−4^ M) solutions in ethanol between 700 and 200 nm at 298 K. As seen, the unbound azo ligand has two separate zones of absorption. The first band at 225 nm could be due to the π-π* transition between the antipyrine and benzene rings. The second band, visible at 365 nm, is related to the n-π* electronic transition (Supplementary Figure S3). Specifically, the n-π* transition band was observed at 346–353 nm, while the π-π* transition band was discovered at 271–275 nm. This demonstrates the ligand-metal ion coupling of the azo dye [38,39].

#### 3.2.7. Molecular Electronic Transitions and Magnetic Moment Measurements

For Cr(III) metal chelate as hexa-coordinated, there were three spins permitted for transitions ^4^A_2_g(F) to^4^ T_1_g(P), ^4^A_2_g(F) to ^4^T_2_g(F), and ^4^A_2_g(F) to ^4^T_2_g(F). These spins allowed transitions in the spectrum of diffused reflectance of Cr(III) chelate at 22,624, 19,436, and 17,361 cm^−1^. The chelate’s stated electronic spectrum is reasonably consistent with those in the literature. The magnetization was determined to be 3.81 B.M. at 298 K, which is in line with what is predicted for octahedral Cr(III) chelate [40].

Three weak bands at 19,950, 21,260, and 25,478 cm^−1^ in the spectrum of diffused reflectance of the Mn(II) metal chelate can be attributed to the transitions ^6^A_1_g to T_2_g (G), ^6^A_1_g to ^5^T_1_g, and charge transfer, correspondingly. The μeff suggested an octahedral geometry. value of 5.51 BM for the manganese(II) metal chelate [41,42].

The Fe(III) metal chelate’s μ_eff_. value was determined to be 5.05 BM, as predicted by the octahedral geometry surrounding the Iron(III) ions [43]. Three bands distinguished the spectrum of reflectance for the Ni(II) metal chelate in the areas ^3^A_2_g(F to ^3^T_2_g(F)(υ1) (13,280 cm^−1^), ^3^A_2_g(F) to ^3^T_1_g(F)(υ_2_) (18,580 cm^−1^) and ^3^A^2^g(F) to ^3^T_1_g(P)(υ_3_) (27,625 cm^−1^) transition in an idealized octahedral geometry.

The μ_eff_. of the Co(II) metal chelate was 5.18 B.M., which correlates to three single electrons. The diffused reflectance spectrum of the Cobalt (II) metal chelate displayed three bands at 10,690 cm^−1^, 15,187 cm^−1^ and 25,051 cm^−1^. The transitions ^4^T_1_g to ^4^T^2^g(F)(ν_1_), ^4^T_1_g to ^4^A_2_g(F), and ^4^T_1_g to T_1_g(P), correspondingly, may be attributed to these bands. Band positions suggested that the Co(II) complex’s an octahedral shape [44,45].

The high spin Ni(II) metal chelate had a μ_eff_. value of 3.76 B.M. at 298 K, indicating that it was an octahedral geometry [46]. Its reflectance spectrum displayed three bands at (13,455 cm^−1^): ^3^A_2_g to ^3^T_2_g; (16,253 cm^−1^): ^3^A_2_g to ^3^T_1_g(F); and (22,056 cm^−1^): ^3^A_2_g to ^3^T_1_g(P). Additionally, a band at 26,333 cm^_1^ was detected in the spectrum, which may be related to the charge transfer of ligand to metal.

The reflectance spectrum for Copper(II) metal chelate showed three sholder bands at 18,950, 21,260, and 24,478 cm^−1^, which could be attributed to ^6^A_1_g to T_2_g (G), ^6^A_1_g to ^5^T_1_g transitions and charge transfer, correspondingly. The μ_eff_. value of 1.79 BM for Cu(II) metal chelate and the electronic transitions suggested an octahedral shape for the complex [47].

An octahedral shape was suggested for the Zn(II) and Cd(II) metal chelates based on their empirical formulae, which were diamagnetic.

#### 3.2.8. Analysis of Thermal Findings

The thermal stability of the azo dye ligand and its metal chelates was inspected using the TG curve (Supplementary Figure S4). Data in Supplementary Table S1 show the disintegration of the ligand and its metal chelates.

The TG curve of the azo dye ligand exhibited three mass loss steps. The first step has a temperature range from 25 to 305 °C anticipating a mass disposal of 28.48% (computed loss of mass = 28.79%), which corresponds to the disposal of C_6_H_7_N. The second step takes place in the range of 305–410 °C with anticipated mass disposal of 9.88% corresponding to C_2_H_6_ molecules. Finally, the last step finished at the temperature between 410–1000 °C with anticipated mass disposal of 61.91%, corresponding to C_9_H_4_N_4_O.

The curve of TG for [Cr(L)(H_2_O)Cl_2_]Cl metal chelate displayed two instances of weight decrease. The first stage of decomposition occurred in the range of 45 to 495 °C, with a max.of 250 °C, and relates to the disposal of H_2_O molecules of coordination, C_6_H_6,_ and chlorine. As in the next step, the mass loss stage (270–800 °C) was due to the ligand’s partial degradation (C_11_H_16_N_5_ClO_1/2_). The final residue was ½ chromium oxide as the residue of decomposition. The TG curve of the Mn(II) metal chelate with a molecular formula [Mn(L)Cl_2_(H_2_O)]2H_2_O was degraded thermally in three stages. The first step occurred within the range of the temperature from 45 to 235 °C, anticipating mass disposal of 7.11% (computed loss of mass = 7.17%), matched with the disposal of coordinated water molecules. The second step takes place in the range of 235 to 475 °C with anticipated mass disposal of 24.54%, corresponding to the disposal of H_2_O, C_2_H_6,_ and Cl_2_ molecules. The last step occurred at the temperature from 475 to 1000 °C, anticipating mass disposal of45.95%, corresponding to disposal of C_15_H_13_N_5_O and MnO as a residue.

It was noted from the TG-DTG curve of [Fe(L)Cl_2_(H_2_O)]Cl.H_2_O metal chelate that the weight loss percentage happened in three stages at 45–1000 °C. In the first step, with a maximum of DTG at 118 °C, 3.51% (theoretically calculated: 3.45%), there was a weight decrease in the temperature range of 45 to 170 °C, which indicated the disposal of the H_2_O molecule of hydration [48]. The second decay stage occurs in the vicinity of 400 to 700 °C, corresponding to the disposal of H_2_O, HCl and Cl2 molecules with anticipated mass disposal of 23.82% (theoretically computed at 23.83%). The azo bonds in the metal chelates under investigation were destroyed when the temperature exceeded 260 °C [49]. The third step of 56.67% (theoretically calculated 56.76%) weight loss was noted in the temperature range of 400 to 1000 °C in the TG curve, which showed the degradation of the C_17_H_15_N_5_O_1/2_ molecule and remaining ½Fe_2_O_3_ as residue [50].

For [Co(L)Cl_2_(H_2_O)]H_2_O metal chelate, the mass disposal of 21.25% (computed 21.72%) within the temperature range of 45 to 215 °C can be assigned to the disposal of two molecules of water and Cl_2_ gas. At the temperature between 215–1000 °C, the mass disposal of 63.14% (calc. 62.90%) was attributed to the disposal of the C_17_H_17_N_5_O molecule. The decomposition was completed, leading to the formation of the nickel oxide (CoO) residue [51].

TG curve of [Ni(L)Cl_2_(H_2_O)] metal chelate exhibited that weight disposal of 18.72% (computed 19.01%) occurred in the temperature range of 45–220 °C is attributable to the mass disposal of H_2_O and Cl_2_. The second step indicated that weight disposal of 13.37% (computed 13.03%) occurred in the temperature range of 220–350 °C, with a max. of DTG at 250 °C, and corresponded to the removal of C_2_H_9_NO. The third step corresponds to mass disposal of 52.56% (calc. 52.13%). In conjunction with this step, the ligand completely decomposes (C_15_H_8_N_4_O) and forms NiO as a residue.

The TG thermogram of [Cu(L)Cl(H_2_O)_2_]Cl metal chelate demonstrated that the decomposition occurred in three stages. The first step within the temperature range of 40 to 250 °C with a maximum of 150 °C may be assigned to the disposal of two H_2_O molecules with a loss of mass of 7.25% (computed = 7.33%). The second degradation step, at 250–485 °C with a maximum of 370 °C, includes disposal of two molecule Cl_2_ gas, with a loss of mass of 14.10% (computed = 14.3%). Finally, the third decomposition step occurred within a temperature range of 285 to 1000 °C with a maximum of 670 °C. The ligand completely evaporates during this stage (C_17_H_17_N_5_O_2_) and produces leftover cupric oxide (CuO).

Zn(II) complex showed TG curves in the temperature range of 45–165 °C disposal of 2H_2_O hydrated water molecules. The second stage is related to the disposal of HCl and coordination water (165–440 °C) at a maximum of 280 °C due to mass disposal of 10.07% (computed 10.53%). The third stage showed the removal of C_17_H_16_N_5_ClO in the temperature range (440–800 °C) relating to the disposal of 66.88 (computed 66.35%). The remaining ligand’s breakdown, which leaves behind zinc oxide residue, causes final weight losses [52].

The TG curve for [Cd(L)Cl_2_(H_2_O)] metal chelate exhibits two separate weight losses. The first step of decomposition occurs within the range of 45 to 520 °C, with a maximum at 250 °C, and corresponds to the disposal of H_2_O, Cl_2_, C_6_H_6_NO, C_6_H_6_, Cl_2,_ corresponding to the removal of 36.91 (computed 37.56%). As in the next step, the loss of mass (520–1000 °C) is caused by the breakdown of a portion of the ligand (C_11_H_11_N_4_). The final residue is CdO.

### 3.3. Correlation between All Findings for Structural Inspection

**Figure 3:**
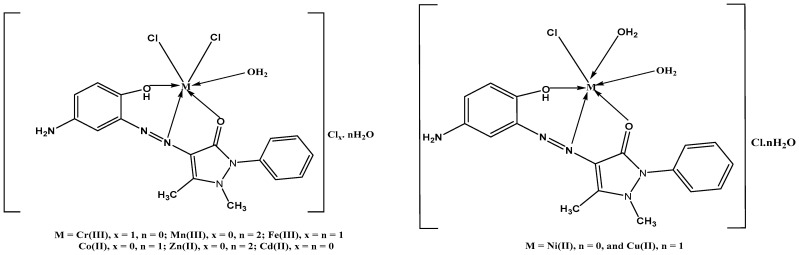
The structure of the L-metal complexes.

From CHN analyses, vibration spectral, molar conductivity, thermal analysis, and mass spectral data, the structures of the metal chelates of the titled ligand with Cr(III), Mn(II), Fe(III), Co(II), Ni(II), Cu(II), Zn(II), and Cd(II) ions were validated. Therefore, from the vibration spectra, it was established that the azo ligand acts as a tri-dentate neutral ligand coupled to the cations via oxygen of the carbonyl group, oxygen of the phenolic group, and one nitrogen of the azo group. The molar conductivity data revealed that the metal chelates were electrolytes, except for Mn^II^, Co^II^, Zn^II^, and Cd^II^. Based on the information mentioned earlier and the magnetic and solid reflectance measurements, an octahedral shape was postulated for the studied metal chelates. In Figure 3, the complexes structural formulas were summed up as follows: [M(L)(H_2_O)Cl_2_]Cl_x_.nH_2_O when M is (Chromium(III), x = 1, n = 0, Manganese(II), x = 0, n = 2, Iron(III), x = n = 1, Cobalt(II), x = 0, n = 1), and Zinc(II), x = 0, n = 2), then [M(L)Cl(H_2_O)_2_]Cl.nH_2_O where M = Nickel(II), n = 0, and Copper(II), n = 1.

### 3.4. DFT Calculations

**Figure 4:**
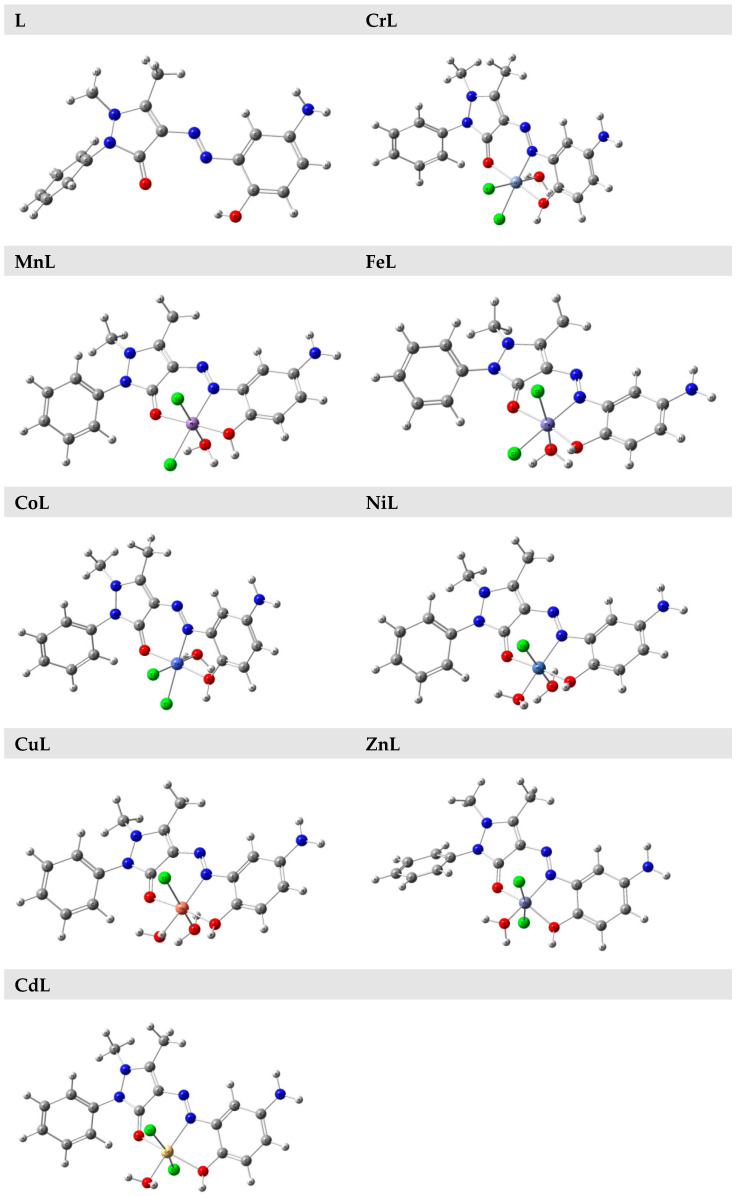
Optimized geometry of the studied compounds.

The B3LYP and 6–311G (d, p) basis sets were used to optimize the geometry of the ligand and its Cr^III^, Mn^II^, Fe^III^, Co^II^, Ni^II^, Cu^II^, Zn^II^, and Cd^II^ metal chelates in the gas phase. Figure 4 displays the L, CrL, MnL, FeL, CoL, NiL, CuL, ZnL, and CdL geometries in their fully optimized and numbered states.

**Figure 5:**
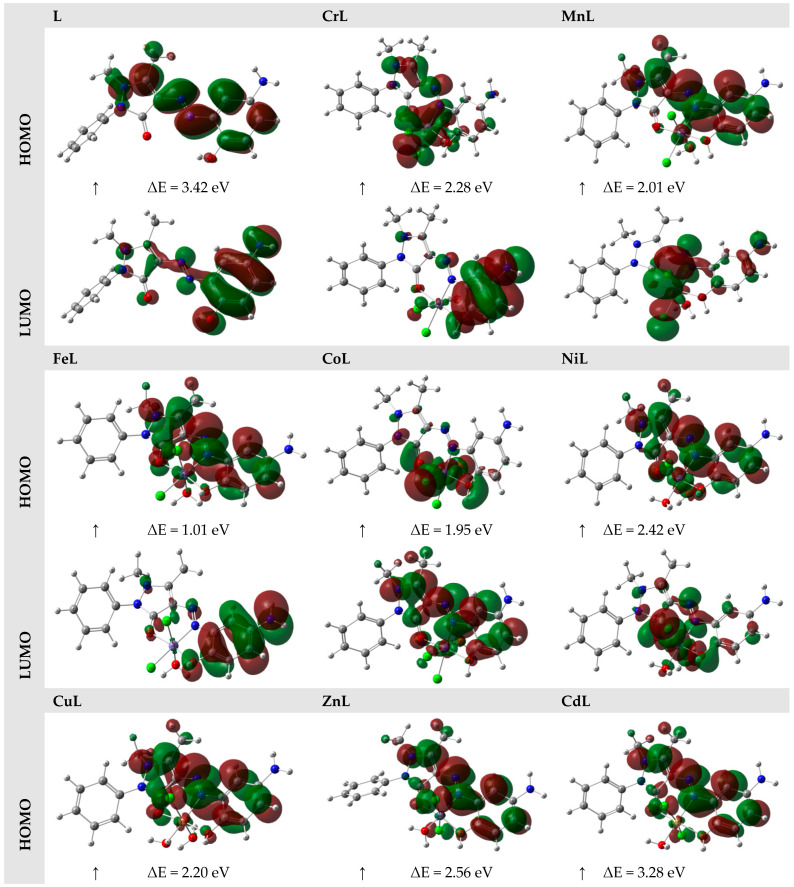

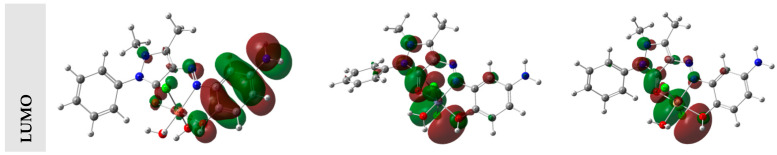
The HOMO and LUMO of the L, CrL, MnL, FeL, Co, NiL, Cu, ZnL, and CdL compounds.

The highest occupied molecular orbital (HOMO) describes the ability of a molecule to release electrons, whereas the smallest vacant molecular orbital describes a molecule’s ability to take electrons (LUMO) [53]. The three-dimensional orbitals that resulted from computing the HOMOs and LUMOs of the compounds using the DFT/B3LYP level of theory are presented in Figure 5, together with their energy values in eV. The HOMO/LUMO of the ligand/metal complexes was concentrated throughout the whole molecule, as seen in Figure 5.

When describing a molecule’s properties, such as chemical stability, the energy difference between HOMO and LUMO is quite helpful [53]. Any molecule’s electron distribution varies less and exhibits low polarization when the HOMO–LUMO energy difference is high. These molecules are referred to as hard molecules. If there is little change in HOMO–LUMO energy, the polarization is strong, the electron distribution is easily steered, and the molecules are referred to as soft molecules. [54].

The HOMO–LUMO energy gap of the ligand and its metal chelates with Cr^III^, Mn^II^, Fe^III^, Co^II^, Ni^II^, Cu^II^, Zn^II^, and Cd^II^ ions, on the other hand, was calculated to be 3.42, 2.28, 2.01, 1.01, 1.95, 2.42, 2.20, and 2.56 eV., respectively. The ligand’s HOMO–LUMO energy gap was greater than the metal complexes. If the examined compounds were ranked according to their energy gaps, L > CdL > ZnL > NiL > CrL > CuL > MnL > CoL > FeL was shown to be the order. The hardness and softness of molecules can be determined based on various factors.

It is crucial to assess the molecules’ HOMO–LUMO energy gaps to provide information. This led to the conclusion that the most challenging and stable molecule was (L). According to calculations, the molecule that was the softest and most reactive was (FeL). As a result, it was found that, among the neutral molecules, (L) was the hardest and most stable, and (FeL) was the softest and most reactive.

Chemical hardness (η), softness (σ), and other parameters are additional chemical descriptors that provide insight into the chemical reactivity of molecules [55,56]. Typically, the HOMO and LUMO energies are used to calculate the values of chemical hardness (η) and softness (σ). Table 1 contains the computed values.

**Figure 6:**
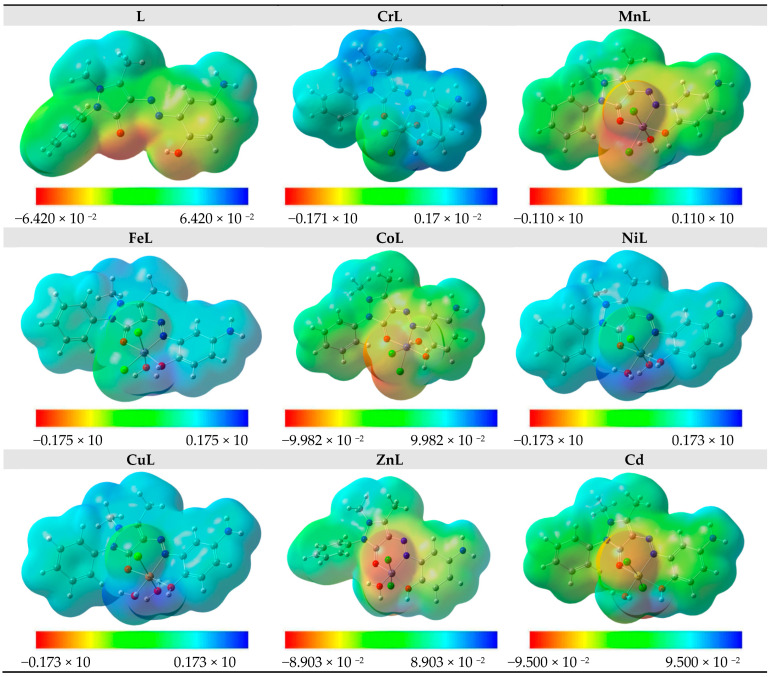
MEP of the studied compounds.

Based on the total electron density surface, the electrostatic potential mapping instantaneously shows the molecular distribution, size, molecular shape, and dipole moments of the molecule’s electrostatic potential (electron + nuclei) [57]. Knowing the relative polarity graphically is useful. The MEP for the ligand and the complexes of CrL, MnL, FeL, CoL, NiL, CuL, ZnL, and CdL are shown in Figure 6. Regions that are electron-rich, electron-deficient, moderately electron-deficient, and neutral are represented on the MEP surface by the colors red, blue, yellow, and green, respectively. In the vicinity of oxygen and chloride, one can find the region with the most negative potential (red) and the most positive charge adjacent to hydrogen atoms.

### 3.5. Biological Activities

The biological properties of the complexes are affected by the ligand’s chelating nature, the type of the donor atoms, the complexes’ overall charge, the metal ion’s nature, the composition of the opposing ions that balance out the complex, and the complex’s geometrical structure [58,59,60]. The antimicrobial action of azo dye compounds may be significantly enhanced by the presence of an azo group with chelating properties. These properties may be used in metal transport across the bacterial membranes, or to attach to the bacterial cells at a specific site, from which it can interfere with their growth [61].

**Figure 7:**
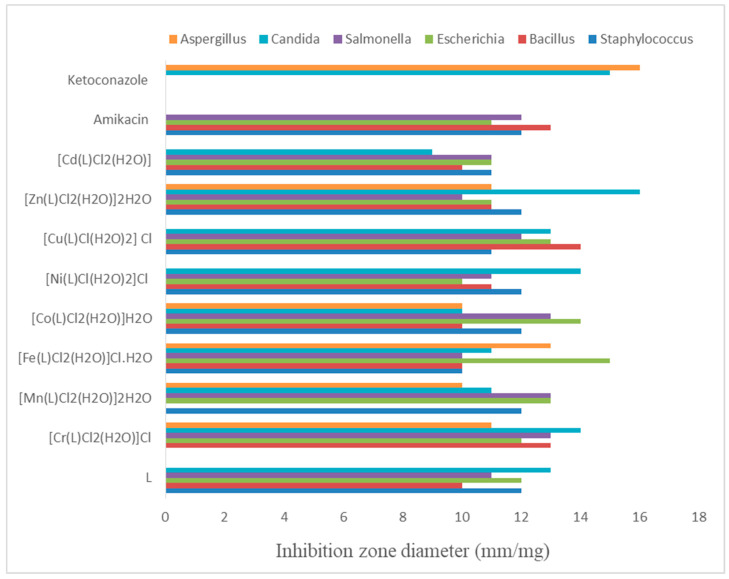
Biological activity of azo dye ligand (L) and its complexes.

Supplementary Table S2 summarizes the antimicrobial characteristics of the azo dye ligand and its metal chelates. The common antibiotics ketoconazole and amikacin were used as the standard antifungal and antibacterial agents, respectively. The results displayed in Figure 7 were obtained by comparing the biological properties of the ligand and its metal chelates. Most complexes were more potent than the free ligand against fungi and bacteria. Furthermore, the Fe(III) metal chelate was the most effective against *A. fumigatus* (lower than ketoconazole standard) and *E. coli* (higher than amikacin standard).

Additionally, the azo dye ligand, Mn(II), Co(II), Ni(II) and Zn(II) metal chelate showed the highest antibacterial potential against *S. aureus* (similar to amikacin standard). Furthermore, the Zn(II), Ni(II) and Cr(III) complexes showed the higher antifungal activity against *C. albicans* and almost higher or similar to the ketoconazole standard. Besides that, Co(II) and Cr(III) complexes demonstrated the most promising antibacterial activity against *Salmonella sp* bacteria and were higher than the antifungal standard. In contrast, the Cu(II) metal chelate showed the highest antibacterial activity against the *B. subtilis* bacteria. In addition, the azo dye ligand, Ni(II), Cu(II) and Cd(II) complexes showed no antifungal activity against *Aspergillus fumigatus*, while the remaining complexes showed remarkable antifungal activity, but lower than the standard. The partial sharing of metal ions’ positive charge with donor groups is the basis for the enhanced antipathogenic action [61,62,63].

It was reported previously [64] that all the compounds have antibacterial activity against Gram positive-bacteria when compared with a gentamicin standard in a similar manner to the cited complexes. The data pointed out that the ligand (HL) and its metal complexes (**1–5**) were found to have no antibacterial activity against Gram-negative bacterium (*P. aeruginosa*), but have antibacterial activity against *E. coli* when compared with an ampicillin standard, which is in contrast to our findings here, where the azo dye ligand and complexes showed remarkable activity. The antifungal activity of HL and its complexes (**1–5**) were reported; they were found to have antifungal activity against *A. fumigatus*, which is in contrast to our findings reported here. The HL ligand, complexes (2) and (4) have no antifungal activity against *C. albicans*, while the complexes (1), (3), and (5) have antifungal activity against *C. albicans,* and the inhibition zone is 19.6, 15.7 and 17.1 mm, respectively, while the azo dye ligand and all metal complexes reported here showed remarkable activity.

The compounds reported previously by researcher [65] exhibited high antimicrobial activity against *E. coli* than *Staphylococcus aureus*. This trend is in similar to our reported data (Supplementary Table S2). It was reported by [65] that the complexes showed higher biological activity than the ligands, as is observed in our study. Thus, the metal coordination enhanced antimicrobial activity [65]. This pattern thus supports the possibility of using these complexes as antimicrobial treatments to treat *E. coli*-related illnesses and/or infections, as surface coating or paint pigments, or as corrosion inhibitors in anti-corrosion paints [65]. The compounds may have the ability to halt or prevent the action of sulphate-reducing bacteria, which starts microbiologically-induced corrosion, based on their antimicrobial activity against anaerobic *E. coli* [65].

The capacity to consider these new compounds and complexes as prospective antibacterial and antitumor agents, as suggested by [38], is indicated by their significant antimicrobial activity against the Gram-negative bacteria *E. coli*.

### 3.6. Molecular Docking

Molecular docking, a crucial technique in the design of the structure, can be used to facilitate and speed up drug discovery by giving researchers information about a virtual screen of the interaction between the target receptor protein and the ligand and predicting the binding affinities and conformations of any species to target proteins.

**Figure 8:**
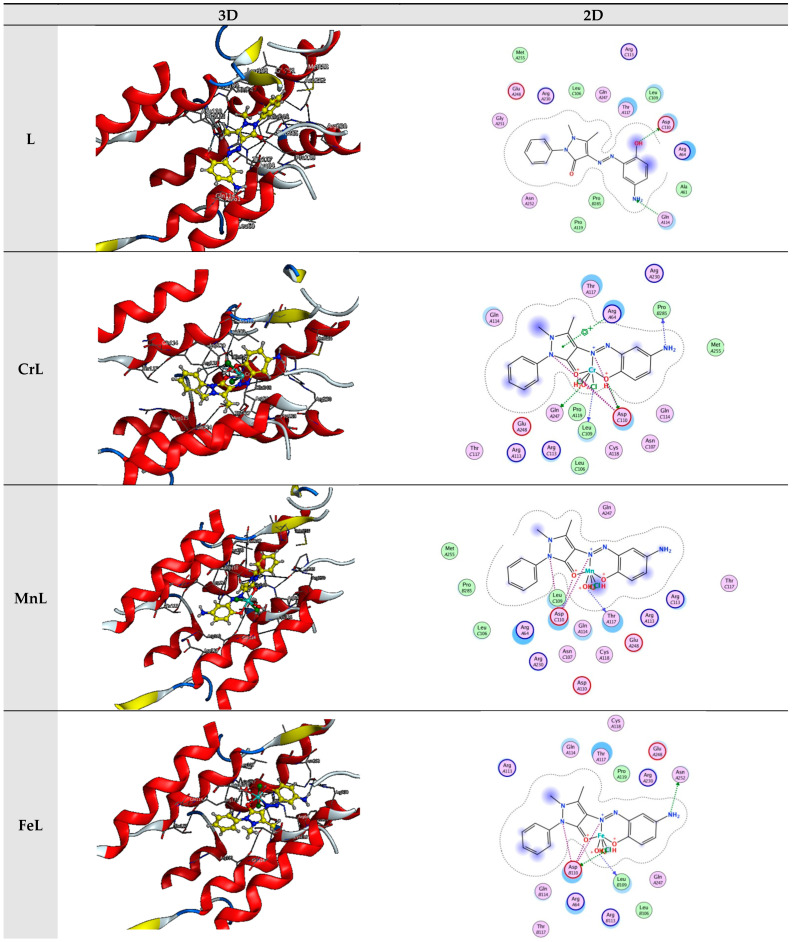

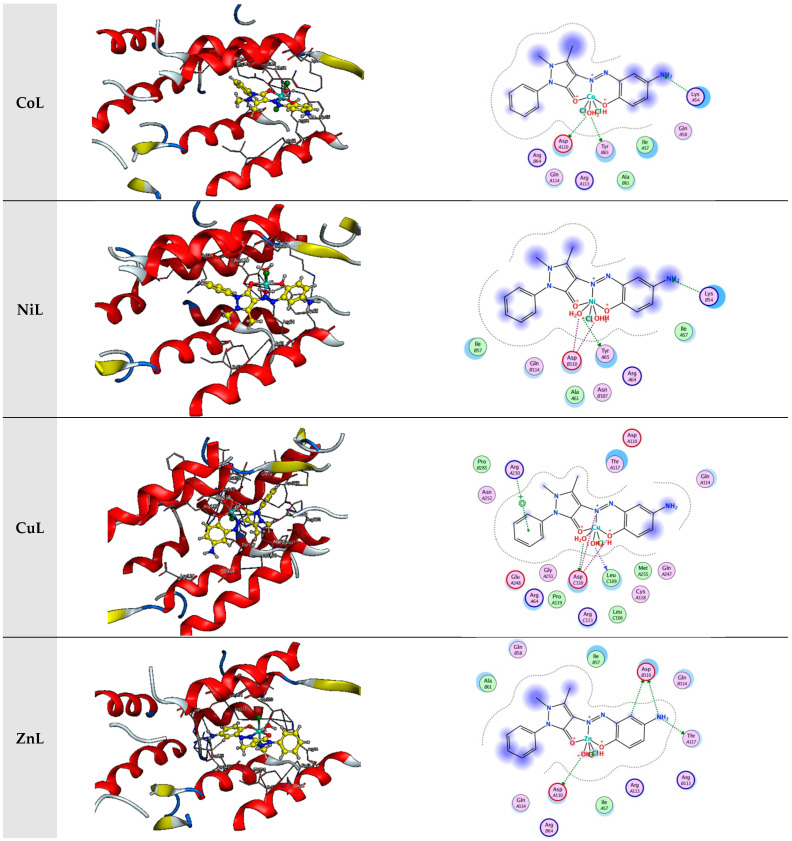

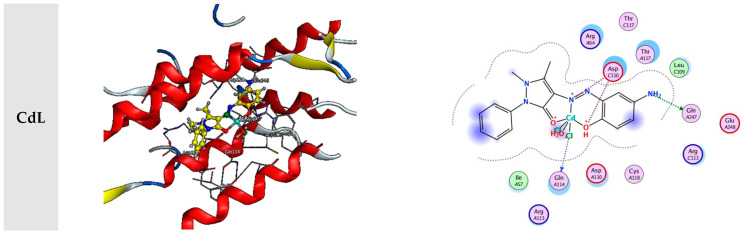
Interaction between the active site of 3t88 with synthesized complexes.

In the present investigation, molecular docking was used to examine the interactions of a few inhibitors with the E’s active site. coli receptor (PDB ID: 3t88). The gathered information is displayed in Figure 8 and Table 2.

The incredible ability to dock toward a targeted protein and the decision of the ligand and target protein’s docking outcomes, which were based on the energy value of associated binding, were both represented by the binding energy’s more considerable negative value. The binding of the ligand and its metal-derived complexes to the *E coli* receptor is mainly mediated by charge and hydrophobic interactions, according to the docking score in Table 2.

The evaluation of the binding scores, resulting from the interaction of all tested compounds with 3t88, revealed that each interaction was exothermic and occurred independently. However, it became apparent that the scores of metal chelates were lower than those of the L ligand when the scores of compounds were compared. This infers that they were more stable than the L-3t88 complex; the complexes formed by binding the complexes to 3t88. L, CrL, MnL, NiL, FeL, CoL, CdL, and ZnL metal complexes’ respective binding scores with 3t88 were −7.82, −7.17, −6.55, −6.50, −6.36, −6.18, −6.11, −5.96, and −5.78 kcal/mol. According to the ratings, the molecule FeL that binds to the 3t88 protein was shown to be the strongest.

Figure 7 depicts the positions the FeL molecule should be in to optimally bind to 3t88 and adhere to the protein’s hydrophobic surface. It was discovered that the ASN 252, LEU 109, and ASP 110 regions of the protein were where the FeL molecule interacted most steadily. Additionally, it was assumed that the protein and shorter hydrogen bond formed this relationship by the FeL complex, as compared to the other compounds, caused the higher binding of the FeL complex to the protein, as opposed to other metal chelates.

## 4. Conclusions

The azo-coupling reaction of 4-amino-antipyrine and 4-aminophenol afforded the azo dye ligand (L). The azo dye structures are verified using IR, 1H NMR, elemental analysis, and mass spectroscopies. The azo dye ligand was reacted with some metal ions to afford a new batch of octahedral-structured transition metal complexes. The structure of the metal chelates was completely elucidated using elemental analysis, infrared, mass, electronic, and ^1^HNMR spectra data, as well as magnetic studies. The IR spectra demonstrated that the chelation mode takes place through the OH (oxygen atom of phenolic), oxygen atoms (of CO group), and the nitrogen atom (of N=N groups). Molar conductivity in ethanol indicates that the Mn^II^, Co^II^, Zn^II,^ and Cd^II^ metal chelates are non-electrolytes. The Cr^III^, Ni^II^, Cu^II,^ and Fe^III^ are electrolytes. The proposed structural formulas of the meta chelates are [M(L)(H_2_O)Cl_2_]Cl_x_.nH_2_O where M = (Cr^III^, x = 1, n = 0, Mn^II^ and Zn^II^, x = 0, n = 2, Fe^III^, x = n = 1, Co^II^, x = 0, n = 1) and Zn^II^, x = 0, n = 2) and [M(L)Cl(H_2_O)_2_]Cl.nH_2_O where M = Ni^II^, n = 0, and Cu^II^, n = 1. Frontier molecular orbitals and optimal geometries were predicted by the DFT with the B3LYP method, using 6-311G (d, p) and LANL2DZ basis sets for the ligand and its metal chelates, respectively. Among all the compounds, FeL complex was the softest and most reactive one, according to the values of the HOMO–LUMO gap. The antimicrobial tests showed that most complexes have higher antifungal activity than the free ligand, and all metal chelates have anti-pathogenic activities behaviors. Additionally, a molecular docking analysis was performed to examine the bonding styles of the molecules produced with the *E. coli* receptor’s active region (PDB ID: 3t88). FeL complex was found to have the lowest binding energy score of all the compounds tested, coming in at −8.82 kcal/mol.

## Figures and Tables

**Table 1:** Chemical descriptors calculated using DFT.

	*E_HOMO_*	*E_LUMO_*	∆*E*	*I*	*A*	*χ*	*CP*	*H*	*σ*	*ω*	*Nu*	ΔN_max_
**L**	−5.27	−1.85	3.42	5.27	1.85	3.56	−3.56	1.71	0.29	3.70	0.27	2.08
**CrL**	−4.57	−2.30	2.28	4.57	2.30	3.44	−3.44	1.14	0.44	5.19	0.19	3.02
**MnL**	−3.77	−1.76	2.01	3.77	1.76	2.77	−2.77	1.01	0.50	3.80	0.26	2.75
**FeL**	−6.68	−5.67	1.01	6.68	5.67	6.18	−6.18	0.50	0.99	37.94	0.03	12.29
**CoL**	−4.56	−2.61	1.95	4.56	2.61	3.59	−3.59	0.97	0.51	6.60	0.15	3.68
**NiL**	−8.81	−6.39	2.42	8.81	6.39	7.60	−7.60	1.21	0.41	23.87	0.04	6.28
**CuL**	−8.66	−6.47	2.20	8.66	6.47	7.57	−7.57	1.10	0.46	26.06	0.04	6.89
**ZnL**	−8.17	−5.61	2.56	8.17	5.61	6.89	−6.89	1.28	0.39	18.51	0.05	5.37
**CdL**	−5.58	−2.30	3.28	5.58	2.30	3.94	−3.94	1.64	0.30	4.73	0.21	2.40

**Table 2:** Docking score obtained and corresponding interaction type.

	Ligand	Receptor	Interaction	Distance	E (kcal/mol)	S (kcal/mol)
**L**	O 8	GLN 114	H-acceptor	3.25	−0.90	−6.78
	N 7	ASP 110	H-donor	2.81	−1.30	
**CrL**	N 7	PRO 285	H-donor	2.75	−3.40	−7.36
	O 8	ASP 110	H-donor	2.92	−3.90	
	O 43	GLN 247	H-donor	2.99	−3.70	
	CL 47	LEU 109	H-donor	3.09	−0.30	
	O 8	ASP 110	ionic	2.92	−5.00	
	O 8	ASP 110	ionic	3.34	−2.50	
	N 10	ASP 110	ionic	3.81	−0.90	
	N 14	ASP 110	ionic	2.98	−4.60	
	O 16	ASP 110	ionic	3.49	−1.90	
	5-ring	ARG 64	pi-cation	3.88	−1.00	
**MnL**	O 43	THR 117	H-donor	2.73	−1.60	−7.55
	N 10	ASP 110	ionic	3.04	−4.20	
	N 14	ASP 110	ionic	3.77	−1.00	
	O 16	ASP 110	ionic	2.67	−7.10	
	O 16	ASP 110	ionic	3.72	−1.20	
**FeL**	N 7	ASN 252	H-donor	2.42	−1.00	−8.82
	O 43	LEU 109	H-donor	2.62	−2.40	
	CL 46	ASP 110	H-donor	2.87	−0.20	
	N 10	ASP 110	ionic	2.79	−1.00	
	N 14	ASP 110	ionic	2.84	−5.60	
	O 16	ASP 110	ionic	3.01	−4.40	
**CoL**	O 43	ASP 110	H-donor	2.80	−3.80	−8.17
	O 43	TYR 65	H-donor	2.69	−3.00	
	N 7	LYS 54	H-acceptor	3.12	−0.20	
	O 43	ASP 110	ionic	2.80	−6.00	
**NiL**	O 47	TYR 65	H-donor	2.86	−6.10	−7.18
	N 7	LYS 54	H-acceptor	3.32	−1.90	
	O 43	ASP 110	ionic	3.44	−2.10	
	O 47	ASP 110	ionic	3.69	−1.20	
**CuL**	O 43	ASP 110	H-donor	2.70	−2.80	−7.50
	O 43	ASP 110	H-donor	2.85	−1.00	
	O 47	LEU 109	H-donor	2.45	−0.60	
	O 8	ASP 110	ionic	2.76	−6.30	
	N 10	ASP 110	ionic	3.00	−4.50	
	O 43	ASP 110	ionic	2.70	−6.90	
	O 43	ASP 110	ionic	2.85	−5.60	
	6-ring	ARG 230	pi-cation	3.31	−0.80	
**ZnL**	C 4	ASP 110	H-donor	3.32	−1.20	−7.11
	N 7	THR 117	H-donor	2.94	−1.60	
	N 7	ASP 110	H-donor	3.46	−0.70	
	O 43	ASP 110	H-donor	2.93	−5.20	
	O 43	ASP 110	ionic	2.93	−5.00	
	O 43	ASP 110	ionic	3.80	−0.90	
**CdL**	N 7	GLN 247	H-donor	3.25	−1.00	−6.96
	CL 47	GLN 114	H-donor	3.23	−2.50	
	O 8	ASP 110	ionic	2.93	−7.40	
	N 10	ASP 110	ionic	3.43	−2.20	

## Data Availability

The raw/processed data generated in this work are available upon request from the corresponding author.

## Supplementary Materials

The following supporting information can be downloaded at: https://www.mdpi.com/article/10.3390/ma16030897/s1, Figure S1: IR spectra of (L) L, (a) L-Cr, (b) L-Mn, (c) L-Fe, (d) L-Co, (e) L-Ni, (f) L-Cu, (g) L-Zn and (h) L-Cd metal complexes; Figure S2: ^1^H-NMR spectra of (a–i) L in DMSO, (b–i) L-Zn(II) in DMSO and (c–i) L-Cd(II) in DMSO; Figure S3. UV-Vis. spectra of (l) L, (a) L-Cr(III), (b) L-Mn(II), (c) L-Fe(III), (d) L-Co(II), (e) L-Ni(II), (f) L-Cu(II), (g) L-Zn(II) and (h) L-Cd(II) complexes; Figure S4. TG Thermograms of (a) L, (b) L-Cr(III), (c) L-Mn(II), (d) L-Fe(III), (e) L-Co(II), (f) L-Ni(II), (g) L-Cu(II), (h) L-Zn(II) and (i) L-Cd(II) complexes. Table S1: Thermodynamic data of the thermal decomposition of azo dye ligand (L) and its metal complexes; Table S2: Biological activity of azo dye ligand (L2) and its metal complexes.
