# Supplementary material for: Design, Synthesis, Spectroscopic Inspection, DFT and Molecular Docking Study of Metal Chelates Incorporating Azo Dye Ligand for Biological Evaluation
Source: Materials (Basel). 2023 Jan 17;16(3):897. doi: 10.3390/ma16030897 (PMC9917733; doi:10.3390/ma16030897)
Supplement: Supplementary file 1 [file materials-16-00897-s001.zip › materials-2089994-supplementary.pdf]

materials

Supplementary Materials

Design, Synthesis, Spectroscopic Inspection, DFT and Molecular Docking Study of Metal Chelates Incorporating Azo Dye Ligand for Biological Evaluation

Mohamed Ali Ibrahim Al-Gaber [1], Hany M. Abd El-Lateef [1,2,*], Mai M. Khalaf [1,2], Saad Shaaban [1,3], Mohamed Shawky [4], Gehad G. Mohamed [4,5], Aly Abdou [2], Mohamed Gouda [1] and Ahmed M. Abu-Dief [2,6,*]

[1] Department of Chemistry, College of Science, King Faisal University, Al-Ahsa 31982, Saudi Arabia
[2] Department of Chemistry, Faculty of Science, Sohag University, Sohag 82534, Egypt
[3] Chemistry Department, Faculty of Science, Mansoura University, Mansoura 35516, Egypt
[4] Chemistry Department, Faculty of Science, Cairo University, Giza 12613, Egypt
[5] Nanoscience Department, Basic and Applied Sciences Institute, Egypt-Japan University of Science and Technology, New Borg El Arab, Alexandria 21934, Egypt
[6] Chemistry Department, College of Science, Taibah University, Medinah 42344, Saudi Arabia
* Correspondence: hmahmed@kfu.edu.sa (H.M.A.E.-L.); amamohammed@taibahu.edu.sa (A.M.A.-D.)

Citation: Al-Gaber, M.A.I.; Abd El-Lateef, H.M.; Khalaf, M.M.; Shaaban, S.; Shawky, M.; Mohamed, G.G.; Abdou, A.; Gouda, M.; Abu-Dief, A.M. Design, Synthesis, Spectroscopic Inspection, DFT and Molecular Docking Study of Metal Chelates Incorporating Azo Dye Ligand for Biological Evaluation. *Materials* **2023**, *16*, 897. https://doi.org/10.3390/ma16030897

Academic Editor: Nicola Margiotta

Received: 25 November 2022
Revised: 9 January 2023
Accepted: 13 January 2023
Published: 17 January 2023

Copyright: © 2023 by the authors. Licensee MDPI, Basel, Switzerland. This article is an open access article distributed under the terms and conditions of the Creative Commons Attribution (CC BY) license (https://creativecommons.org/licenses/by/4.0/).

Chemicals

All chemicals used in this study were of pure grade and of highest purity available and used without further purification. The chemicals used included 4-aminophenol, 4-amino-antipyrine, $CrCl_3.6H_2O$ (Sigma), $MnCl_2.2H_2O$ (Sigma), $NiCl_2.6H_2O$ (BDH), FeCl3.6H2O (Prolabo), $CoCl_2.6H_2O$ (Aldrich), $CuCl_2.2H_2O$ (Merck), $ZnCl_2$ (Strem Chemicals), and $CdCl_2$ (Aldrich). Organic solvents were spectroscopic pure from BDH, smf included ethanol and dimethylformamide. Hydrochloric acid, sodium nitrite and sodium acetate (A.R.) were used.

Measurements

Microanalyses of carbon, hydrogen, and nitrogen were carried out at the Microanalytical Center, Cairo University, Egypt, using CHNS-932 (LECO) Vario Elemental Analyzer. Analyses of the metals followed the dissolution of the solid complexes in concentrated HNO_3, neutralizing the diluted aqueous solutions with ammonia and titrating the metal solutions with EDTA. 1H NMR spectra, as a solution in DMSO-d6, were recorded on a 300 MHz Varian-Oxford Mercury at room temperature, using TMS as an internal standard. Mass spectra were recorded by the EI technique at 70 eV, using a MS-5988 GS-MS Hewlett–Packard instrument at the Microanalytical Center, National Center for Research, Egypt. FT-IR spectra were recorded on a Perkin–Elmer 1650 spectrometer (4000–400 cm^{-1}) as KBr pellets. The electronic spectra were recorded in DMSO at room temperature on Shimadzu UV–Visible mini-1240 spectrophotometer. The molar magnetic susceptibility was measured on powdered samples using the Faraday method. The diamagnetic corrections were made by Pascal's constant, and $Hg[Co(SCN)_4]$ was used as a calibrant. Molar conductivities of 10^{-3} M solutions of the solid complexes in ethanol were measured using a Jenway 4010 conductivity meter. The thermogravimetric analyses (TG and DTG) of the solid complexes were carried out using a Shimadzu TG-50H thermal analyzer in a dynamic nitrogen atmosphere (flow rate 20 mL min^{-1}) with a heating rate of 10 °C min^{-1}. The percentage mass loss was measured from the ambient temperature up to 1000 ^{0}C. Highly sintered α-Al_2O_3 was used as a reference. Diffused reflectance spectra analyses were carried out at the Microanalytical Center, Cairo University, Egypt. The antimicrobial activities were carried out at the Microanalytical Center, Cairo University, Egypt.

Table S1. Thermodynamic data of the thermal decomposition of azo dye ligand (L) and its metal complexes.

Complex	TG Range (°C)	DTG Max (°C)	n*	Mass Loss<br>Loss Found	Total Mass<br>(Calcd) %	Assignment<br>Disposal of	Residues
L	25–305	220	1	28.48(28.79)		- C_6H_7N	
	305–410	371	1	9.88(9.29)		- C_2H_6	
	410–1000	650	1	61.64(61.93)	100 (100)	- $C_9H_4N_4O$	
$[Cr(L)Cl_2(H_2O)]Cl$	45–495	250	1	35.72(35.35)		- H_2O, $C_6H_6Cl_2$	
	495–1000	655	1	50.33(50.75)	86.05(86.10)	- $C_{11}H_{16}N_5ClO_{1/2}$	½Cr_2O_3
$[Mn(L)Cl_2(H_2O)]2H_2O$	45–235	118	1	7.11(7.17)		- $2H_2O$	
	235–475	360	1	24.54(23.50)		- H_2O, C_2H_6, Cl_2	
	475–1000	360	1	54.95(55.57)	86.60(86.24)	- $C_{15}H_{13}N_5O$	MnO
$[Fe(L)Cl_2(H_2O)]Cl.H_2O$	45–170	118	1	3.51(3.45)		- H_2O	
	170–400	289	1	23.82(23.83)		- H_2O, HCl, Cl_2	
	400–1000	566,661	2	56.67(56.76)	84.00(84.04)	- $C_{17}H_{15}N_5$, ½O_2	½Fe_2O_3
$[Co(L)Cl_2(H_2O)]H_2O$	45–215	175	1	21.25(21.72)		- $2H_2O$, Cl_2	
	215–1000	430,810	2	63.14(62.90)	84.39 (84.62)	- $C_{17}H_{17}N_5O$	CoO
$[Ni(L)Cl(H_2O)_2]Cl$	45–220	170	1	18,72(19,01)		- H_2O, Cl_2	
	220–350	250	1	13,37(13,03)		- C_2H_9NO	
	350–1000	470,650	2	52,56(52,13)	84.65(84.17)	- $C_{15}H_8N_4$ O	NiO
$[Cu(L)Cl(H_2O)_2]$ Cl	45–245	185	1	7.32(7.25)		- $2H_2O$	
	245–490	365	1	14.10(14.30)		- Cl_2	CuO
	4901000	660	1	61.65(61.83)	83.07(83.38)	- $C_{17}H_{17}N_5O$	
$[Zn(L)Cl_2(H_2O)]2H_2O$	45–165	95	1	7.23(7.02)		- $2H_2O$	
	165–440	280	1	10.07(10.53)		- HCl, H_2O	ZnO
	440–800	535,605	2	66.88(66.35)	84.18(83.9)	- $C_{17}H_{16}N_5ClO$	
$[Cd(L)Cl_2(H_2O)]$	45–515	250	1	36.91(37.56)		- H_2O, Cl_2, C_6H_6NO	CdO
	515–1000	685	1	38.52(37.94)	75.43(75.50)	- $C_{11}H_{11}N_4$	

Table S2. Biological activity of azo dye ligand (L^2) and its metal complexes.

Sample	Inhibition Zone Diameter ($mm\ mg^{-1}$ sample)					
	Staphylococcus aureus (G^+)	*Bacillus subtilis* (G^+)	*Escherichia coli* (G_-)	*Salmonella*	*Candida albicans (fungus)*	*Aspergillus Fumigatus*
Ligand (L)	12	10	12	11	13	NA
$[Cr(L)Cl_2(H_2O)]Cl$	NA	13	12	13	14	11
$[Mn(L)Cl_2(H_2O)]2H_2O$	12	NA	13	13	11	10
$[Fe(L)Cl_2(H_2O)]Cl.H_2O$	10	10	15	10	11	13
$[Co(L)Cl_2(H_2O)]H_2O$	12	10	14	13	10	10
$[Ni(L)Cl(H_2O)_2]Cl$	12	11	10	11	14	Na
$[Cu(L)Cl(H_2O)_2]Cl$	11	14	13	12	13	Na
$[Zn(L)Cl_2(H_2O)]2H_2O$	12	11	11	10	16	11
$[Cd(L)Cl_2(H_2O)]$	11	10	11	11	9	NA
Amikacin	12	13	11	12	----------	----------
Ketoconazole	-----------	---------	----------	-------	15	16

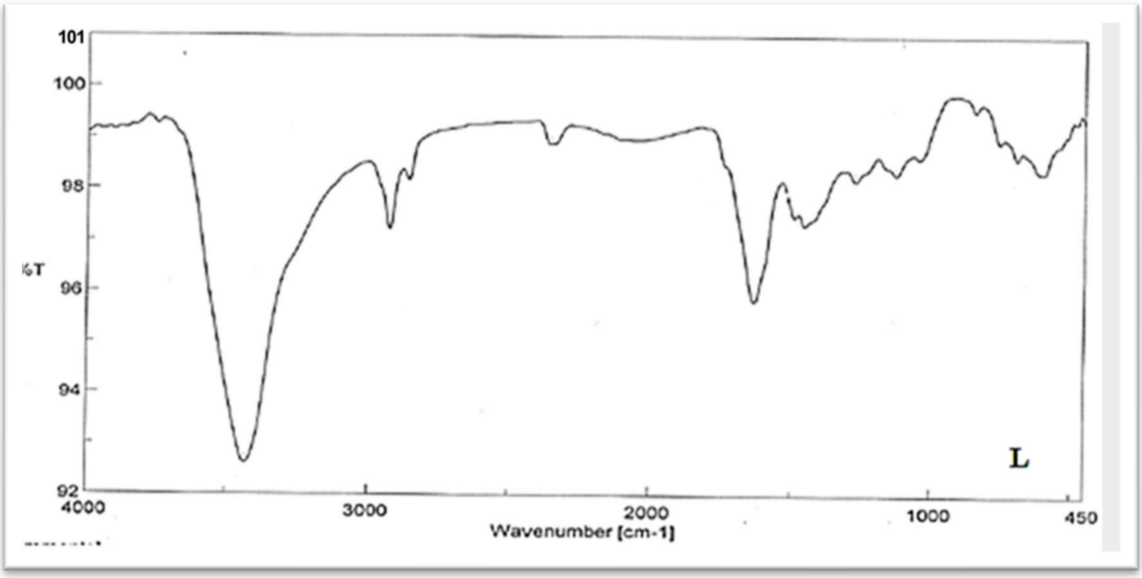

101
100
98
%T
96
94
92
4000
3000
2000
1000
450
Wavenumber [cm-1]
L

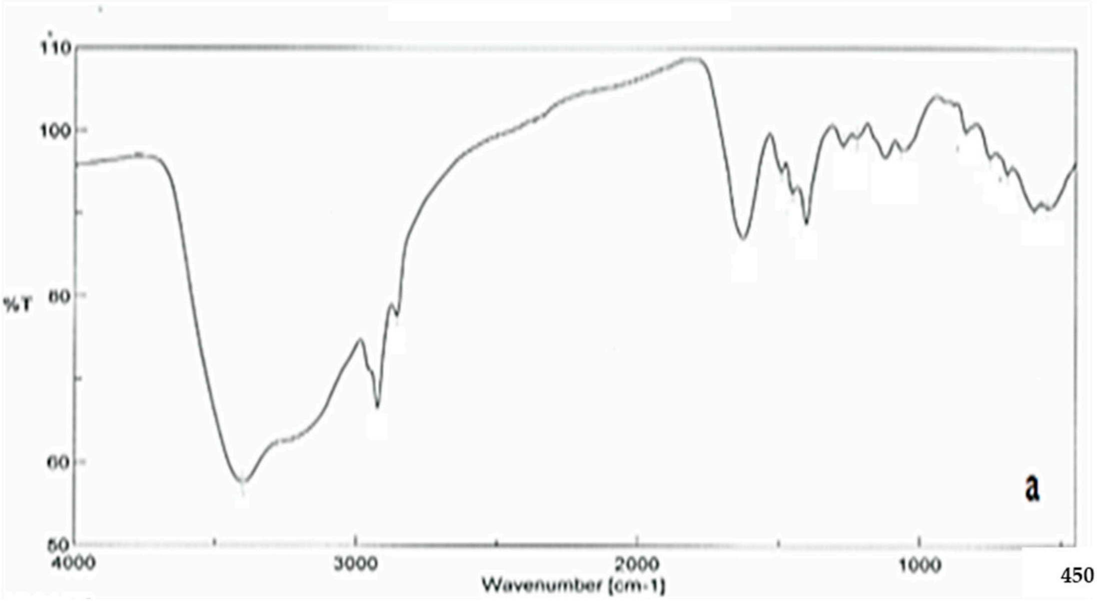

110
100
%T
80
60
50
4000
3000
2000
1000
450
Wavenumber [cm-1]
a

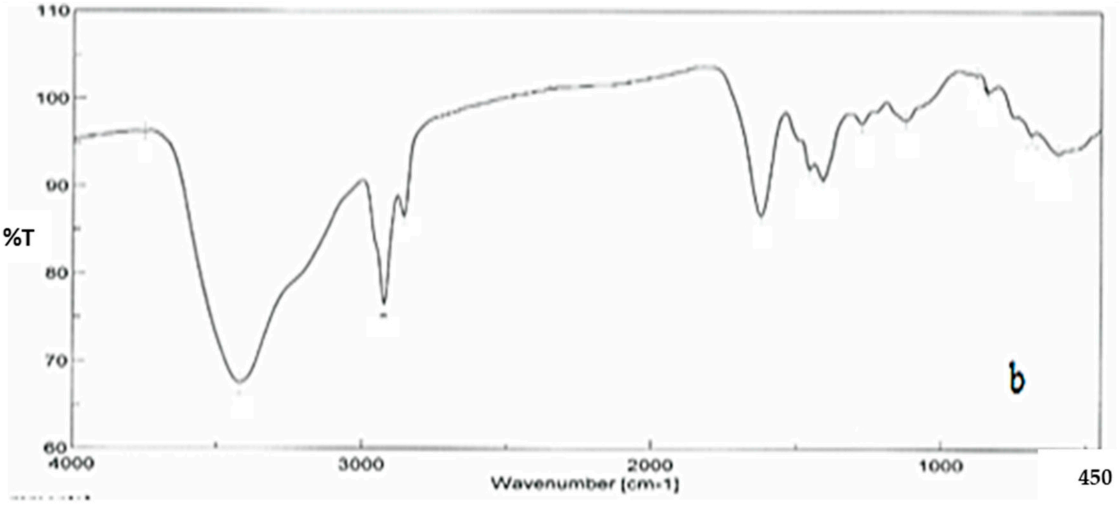
110
100
90
%T
80
70
60
4000
3000
2000
1000
450
Wavenumber [cm-1]
b

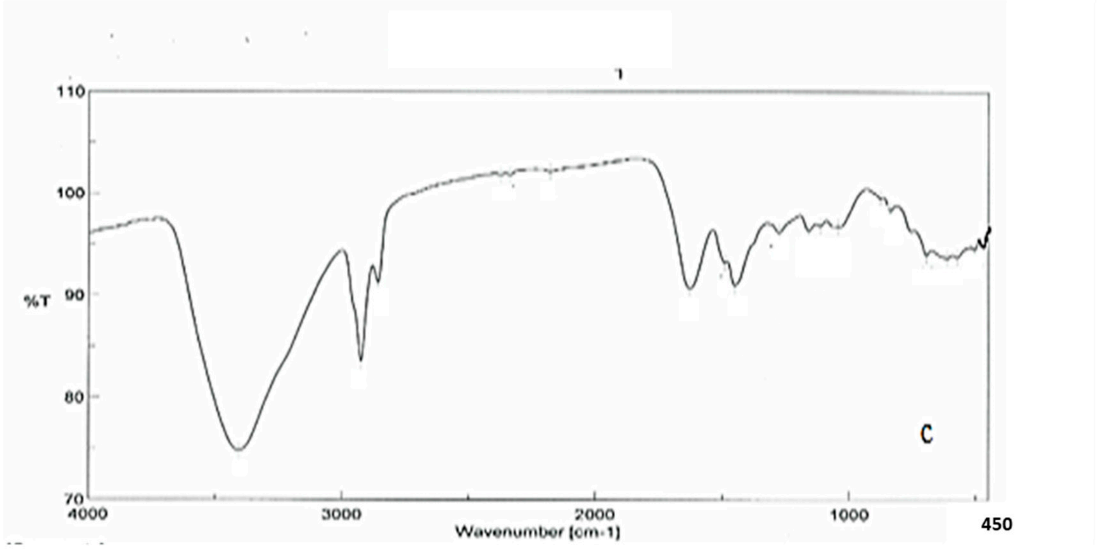
110
100
%T
90
80
70
4000
3000
2000
1000
450
Wavenumber [cm-1]
c

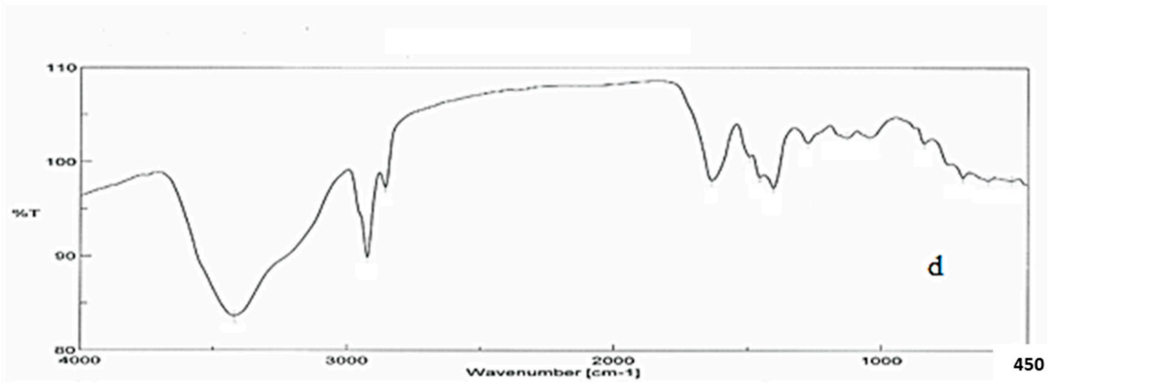
110
100
%T
90
80
4000
3000
2000
1000
450
Wavenumber [cm-1]
d

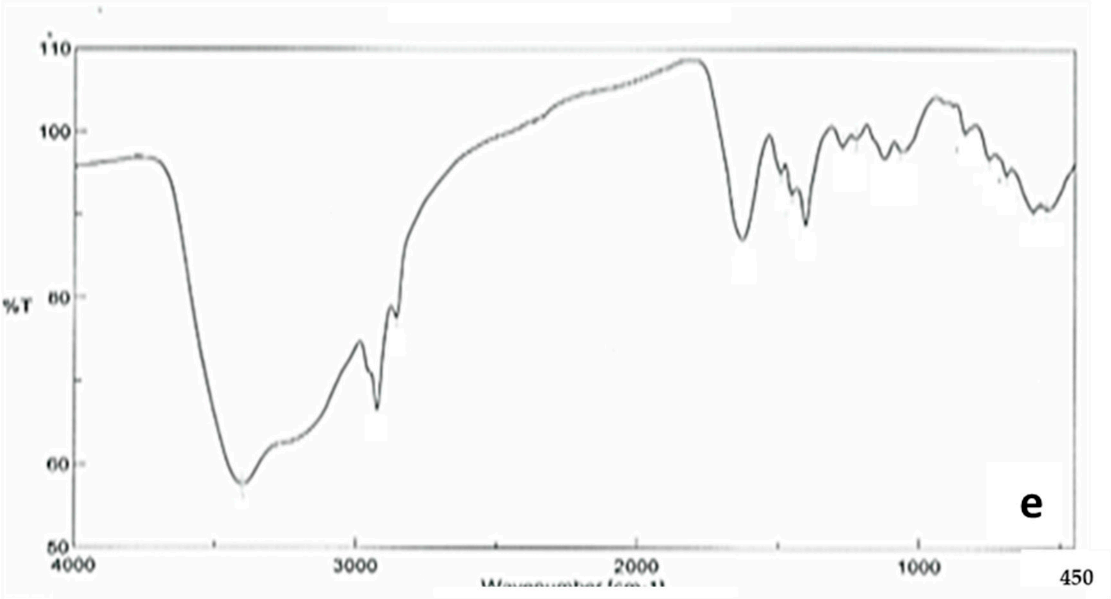
110
100
%T
80
60
50
4000
3000
2000
1000
450
e

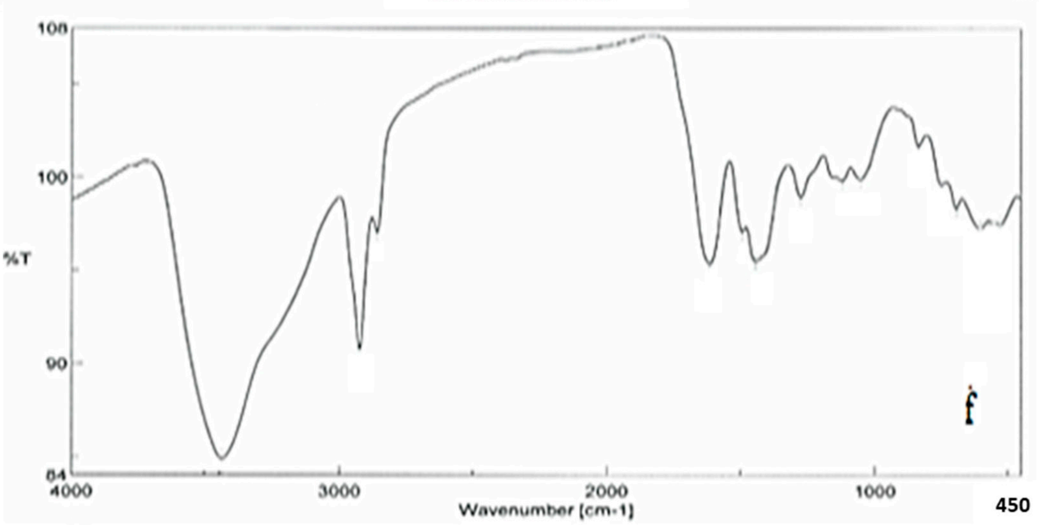
108
100
%T
90
84
4000
3000
2000
1000
450
Wavenumber (cm-1)
f

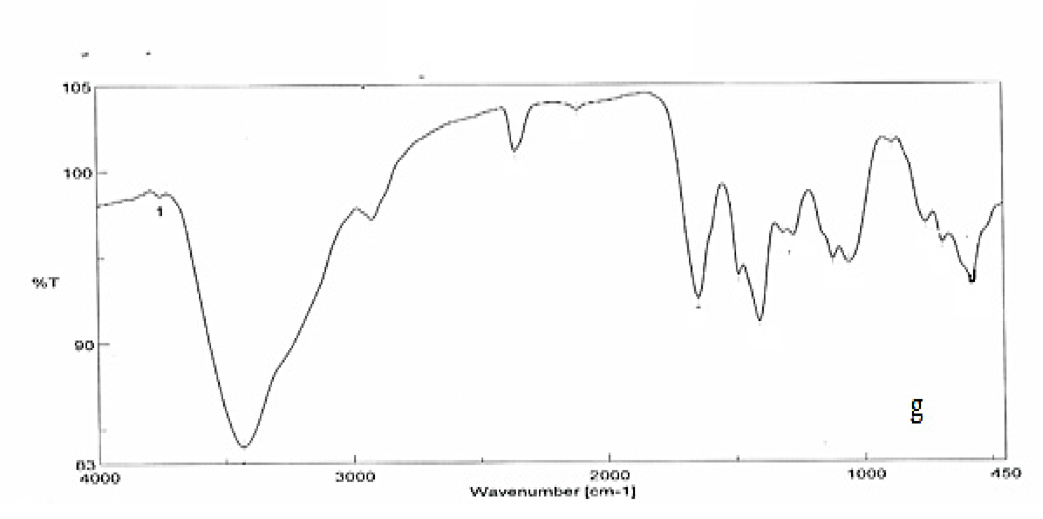
105
100
%T
90
83
4000
3000
2000
1000
450
Wavenumber (cm-1)
g

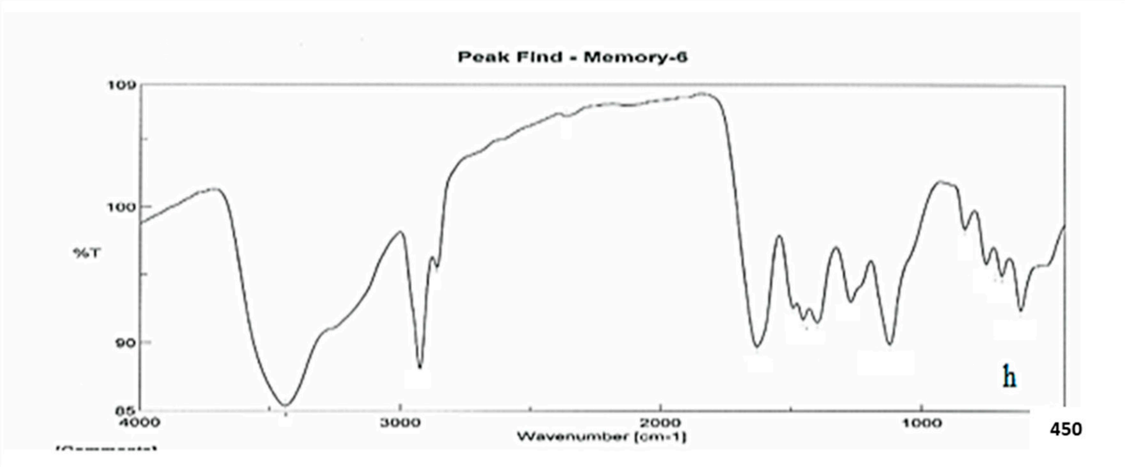

Figure S1. IR spectra of (L) L, (**a**) L-Cr, (**b**) L-Mn, (**c**) L-Fe, (**d**) L-Co, (**e**) L-Ni, (**f**) L-Cu, (**g**) L-Zn and (**h**) L-Cd metal complexes.

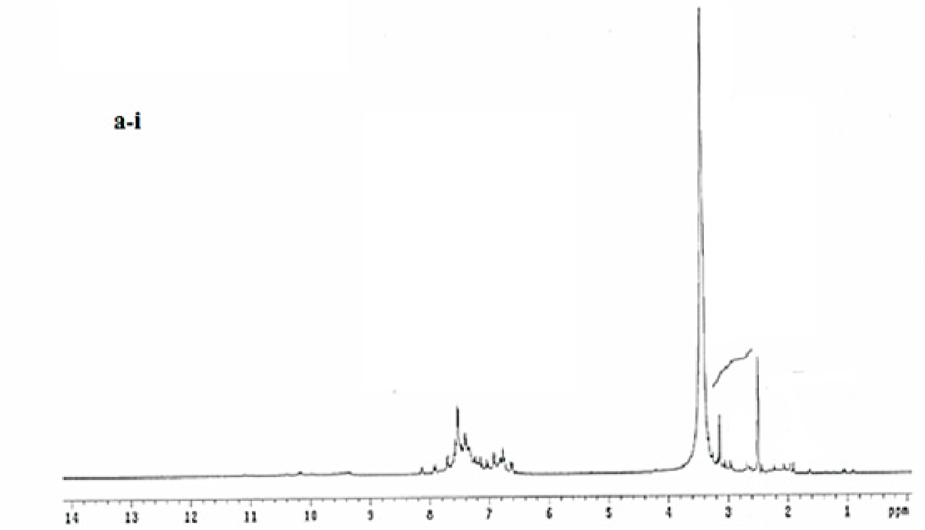

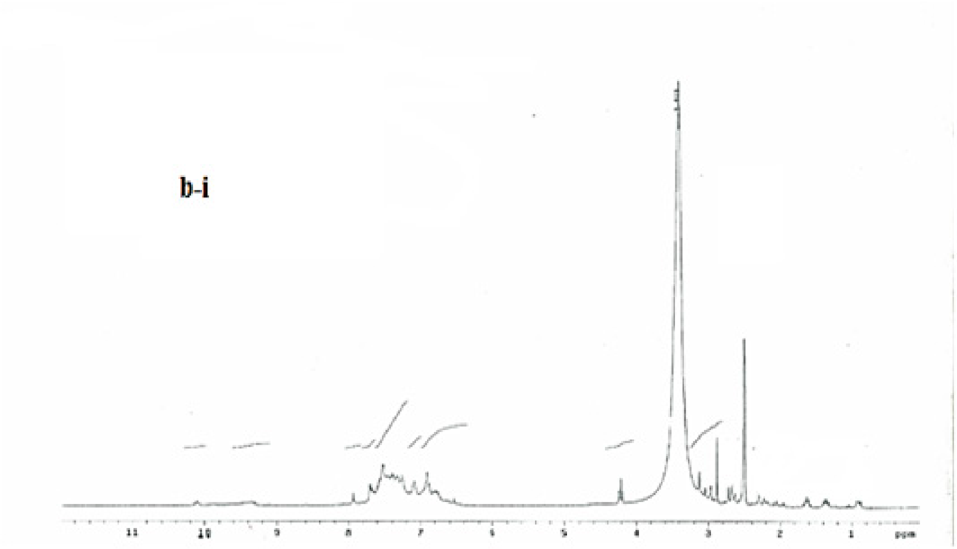

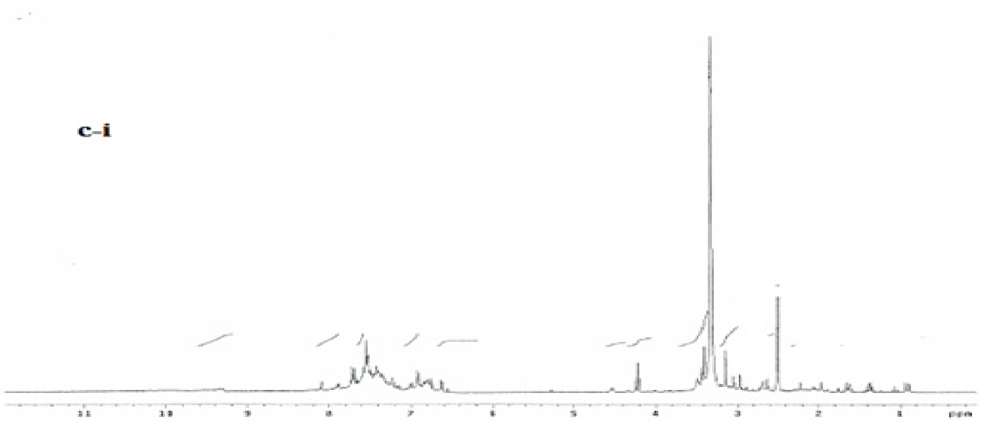

Figure S2. ^{1}H-NMR spectra of (**a-i**) L in DMSO, (**b-i**) L-Zn(II) in DMSO and (**c-i**) L-Cd(II) in DMSO.

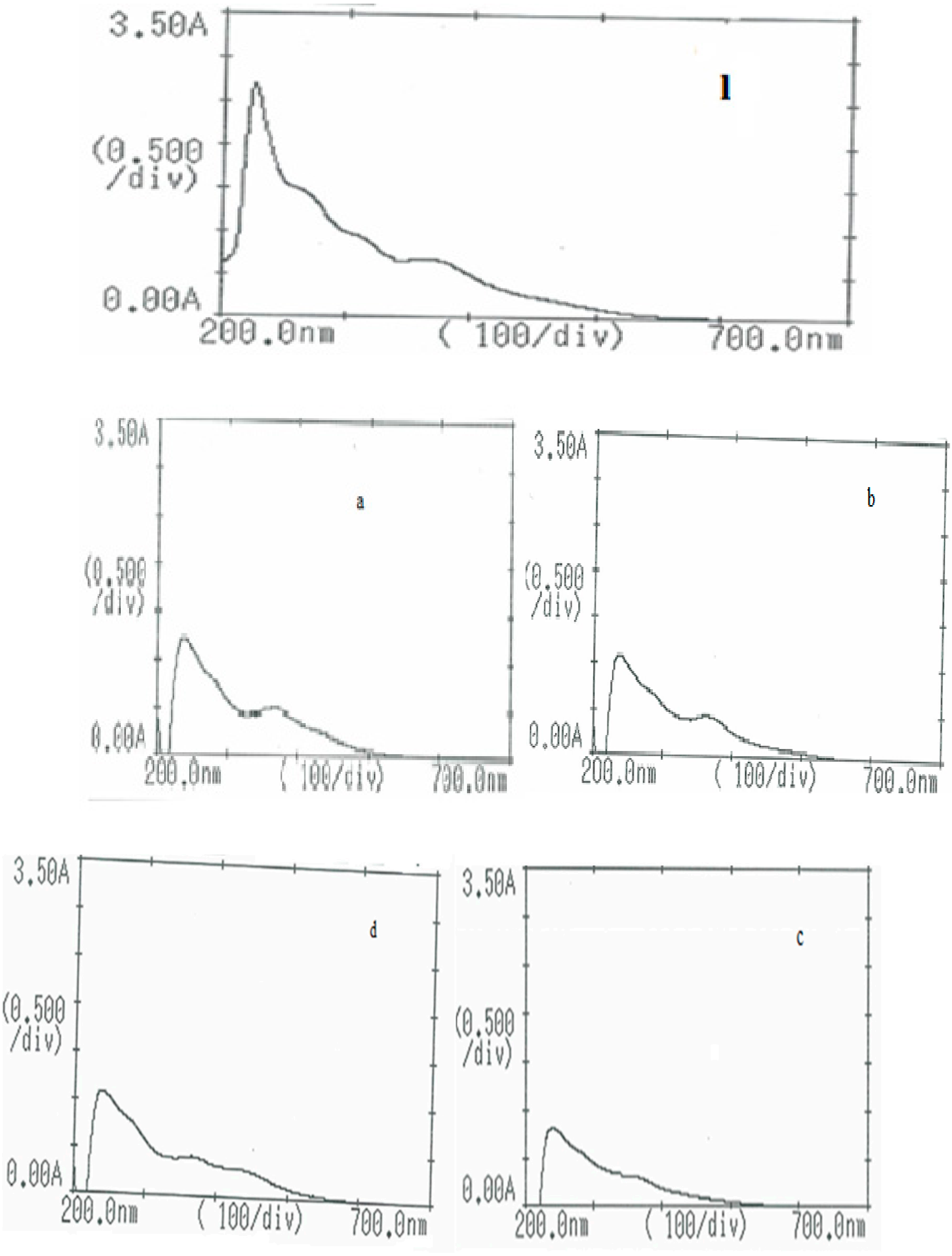
3.50A
(0.500
/div)
0.00A
200.0nm
(100/div)
700.0nm
1
a
b
d
c

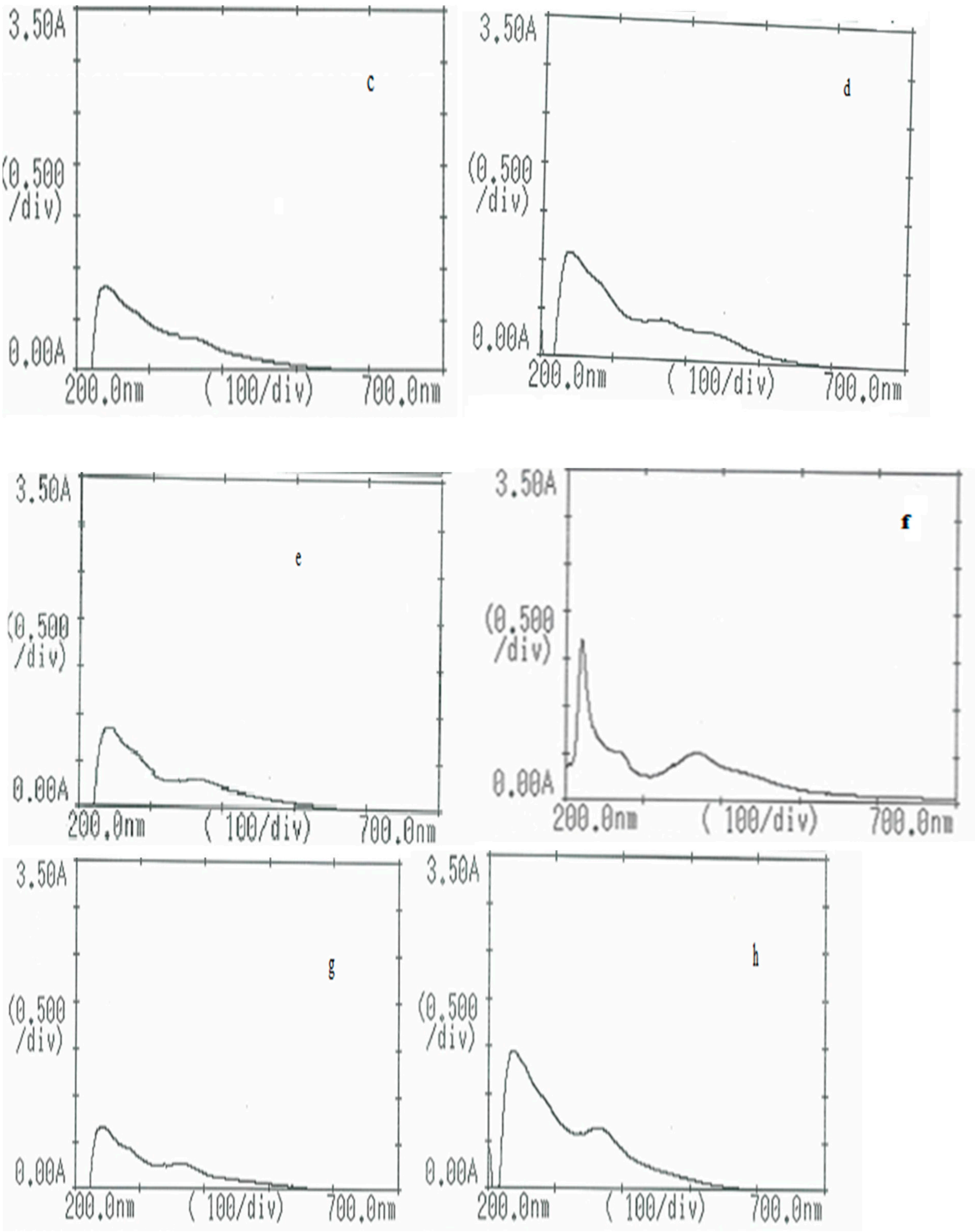

Figure S3. UV-Vis. spectra of (l) L, (**a**) L-Cr(III), (**b**) L-Mn(II), (**c**) L-Fe(III), (**d**) L-Co(II), (**e**) L-Ni(II), (**f**) L-Cu(II), (**g**) L-Zn(II) and (**h**) L-Cd(II) complexes.

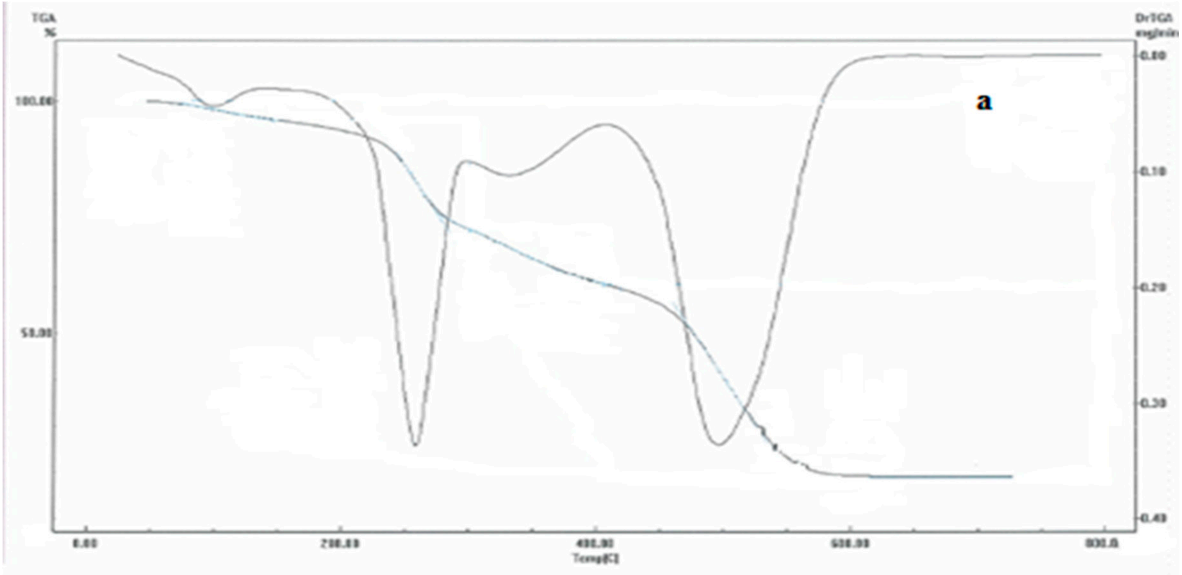
TGA
%
DrTGA
mg/min
a
100.00
50.00
Temp[C]

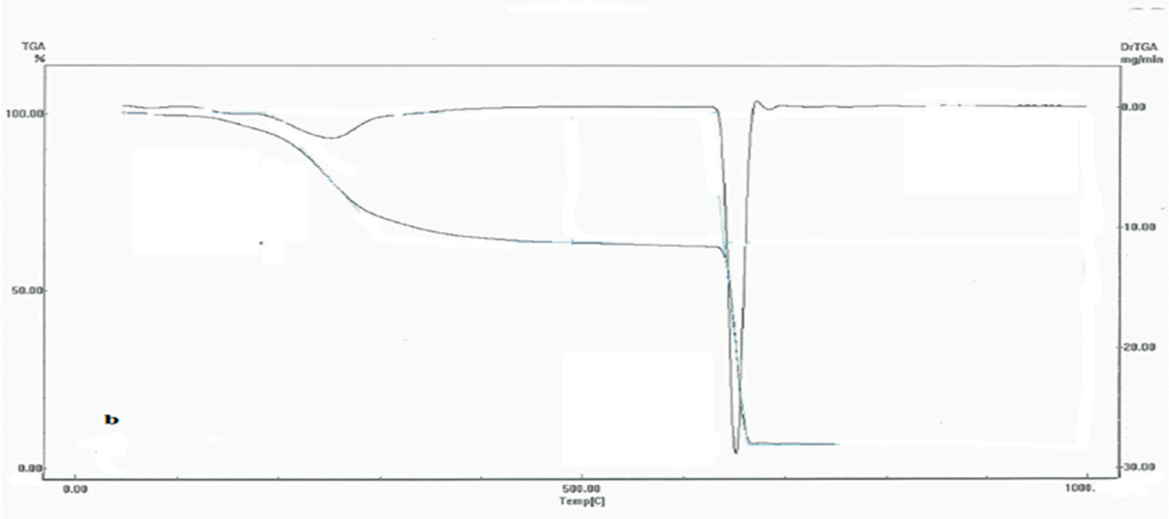
TGA
%
DrTGA
mg/min
100.00
50.00
0.00
0.00
10.00
20.00
30.00
500.00
1000.
Temp[C]
b

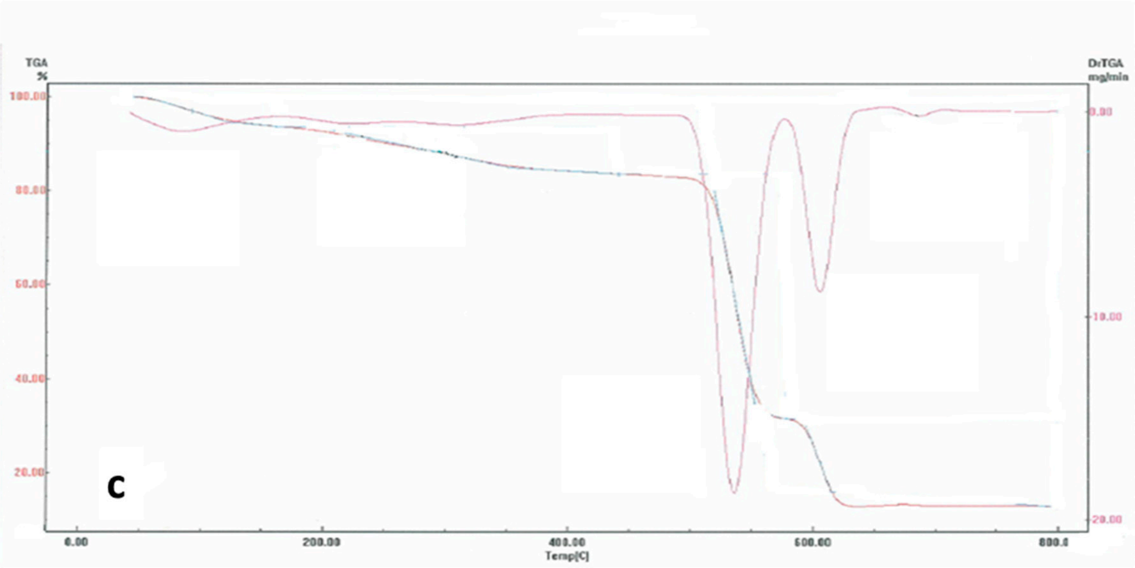
TGA
%
DrTGA
mg/min
100.00
80.00
60.00
40.00
20.00
0.00
-10.00
-20.00
0.00
200.00
400.00
600.00
800.0
Temp[C]
c

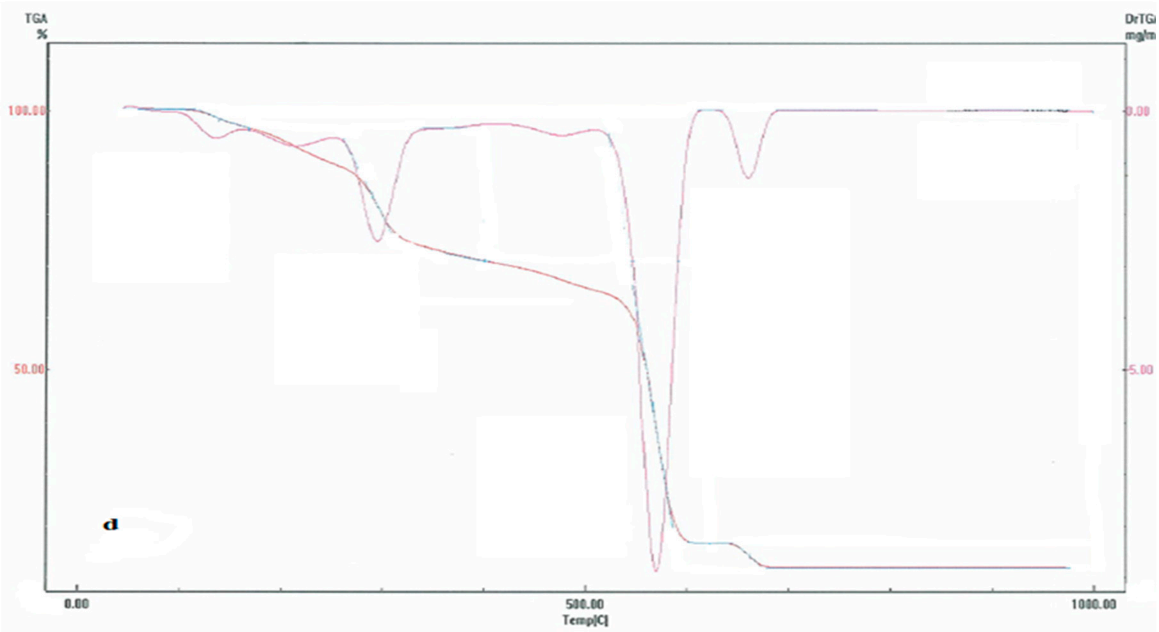
TGA
%
100.00
50.00
0.00
-5.00
0.00
500.00
1000.00
Temp[C]
d

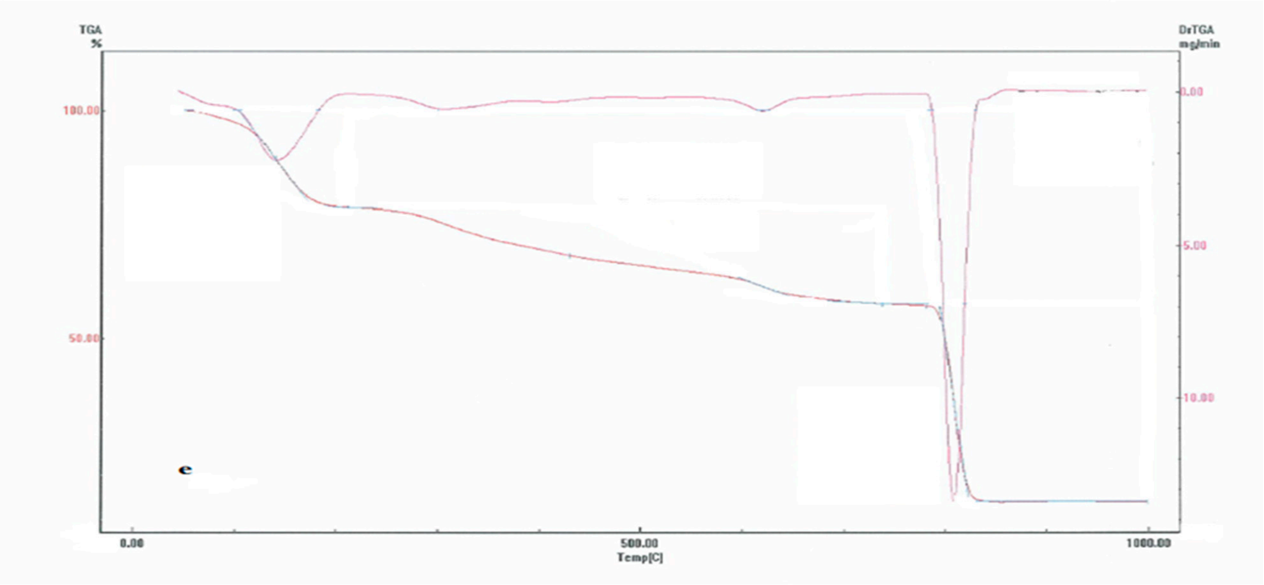
TGA
%
DrTGA
mg/min
100.00
50.00
0.00
-5.00
-10.00
e
0.00
500.00
1000.00
Temp[C]

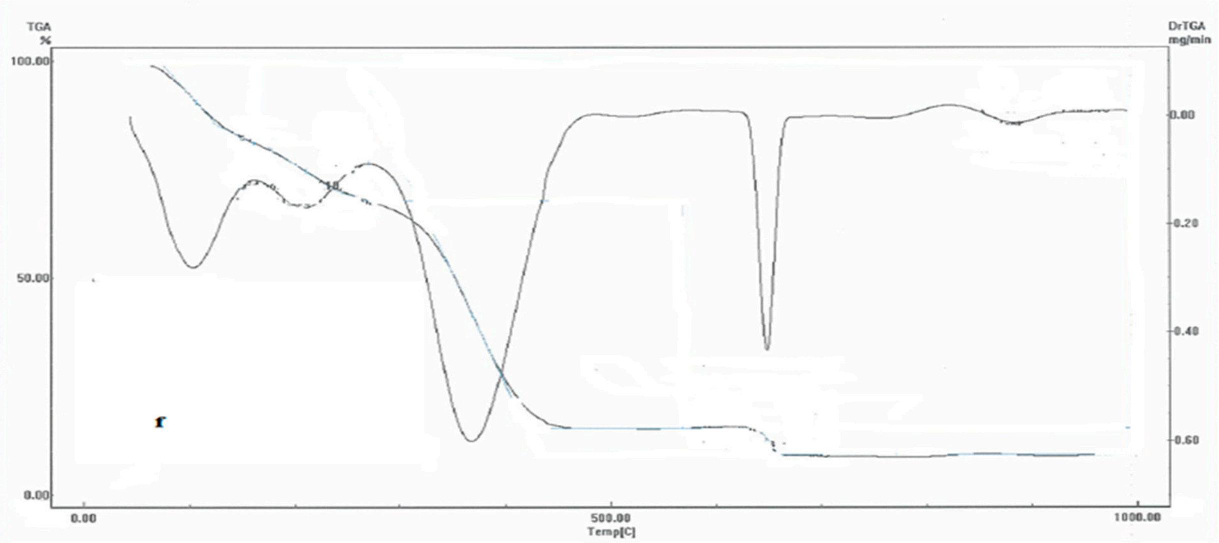
TGA
%
DrTGA
mg/min
100.00
50.00
0.00
0.00
-0.20
-0.40
-0.60
f
0.00
500.00
1000.00
Temp[C]

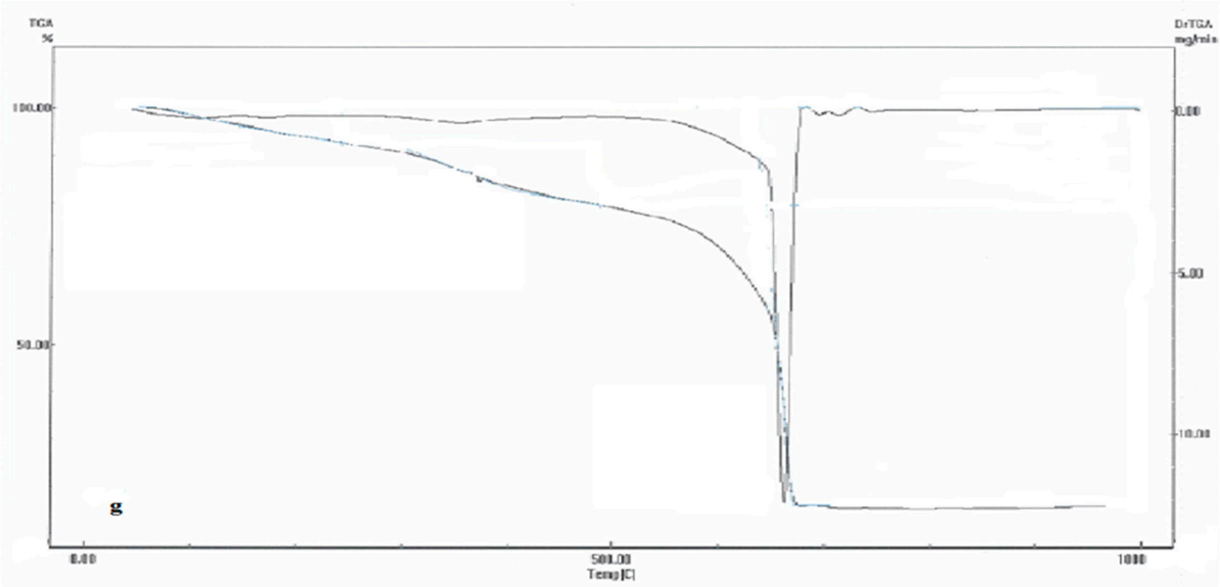
TGA
%
DrTGA
mg/min
100.00
50.00
0.00
-5.00
-10.00
g
0.00
500.00
1000
Temp[C]

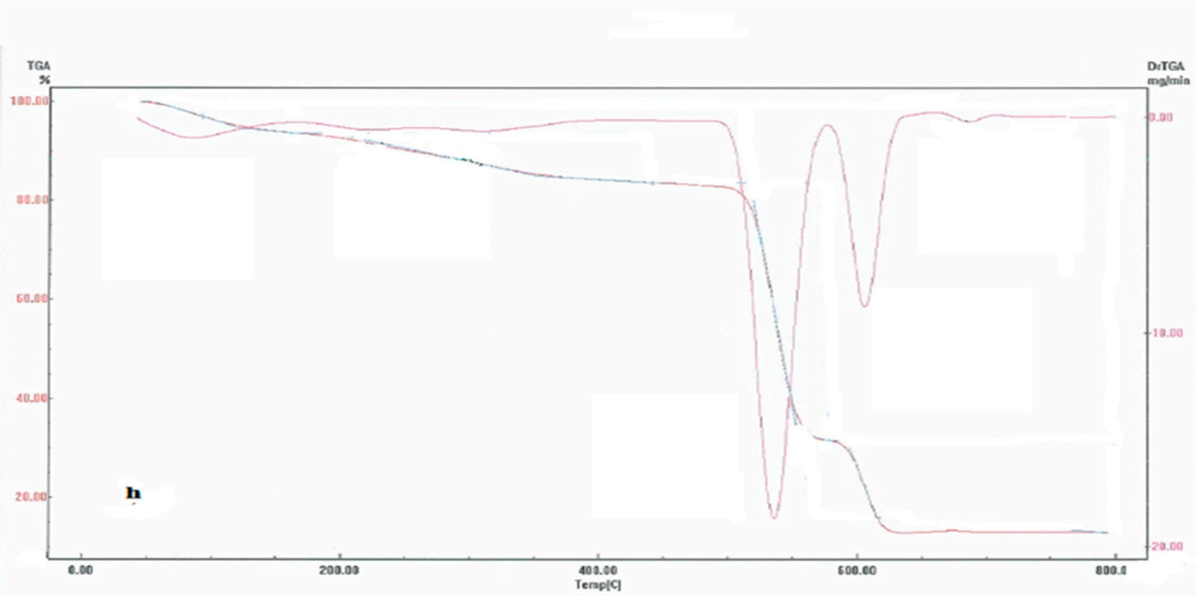
TGA
%
DrTGA
mg/min
100.00
80.00
60.00
40.00
20.00
0.00
-10.00
-20.00
h
0.00
200.00
400.00
600.00
800.0
Temp[C]

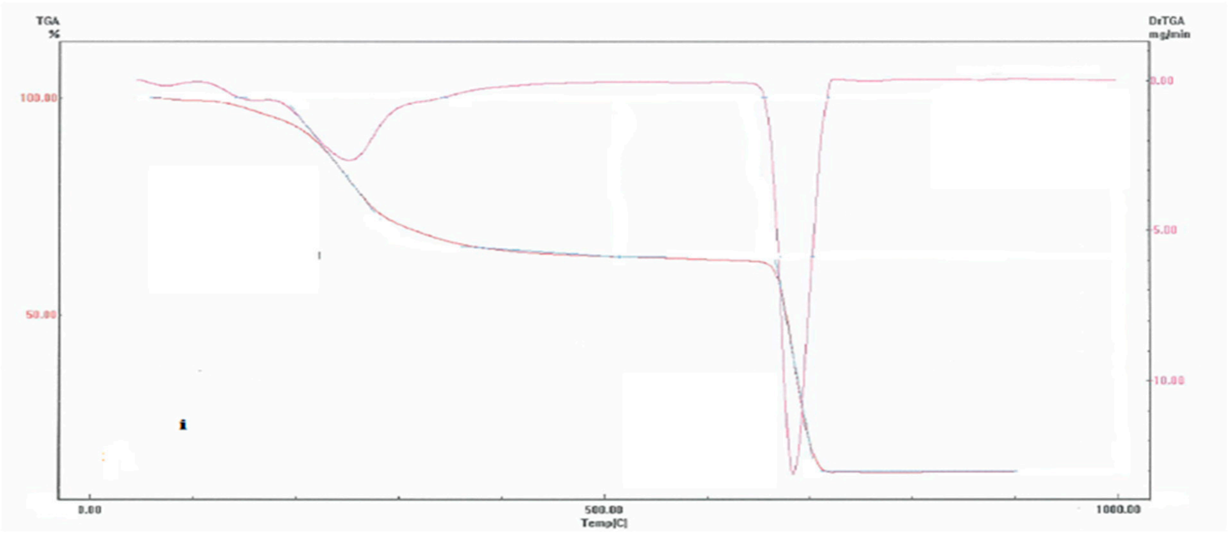

Figure S4. TG Thermograms of (**a**) L, (**b**) L-Cr(III), (**c**) L-Mn(II), (**d**) L-Fe(III), (**e**) L-Co(II), (**f**) L-Ni(II), (**g**) L-Cu(II), (h) L-Zn(II) and (**i**) L-Cd(II) complexes.
